# Bothrops Jararaca Snake Venom Modulates Key Cancer-Related Proteins in Breast Tumor Cell Lines

**DOI:** 10.3390/toxins13080519

**Published:** 2021-07-25

**Authors:** Carolina Yukiko Kisaki, Stephanie Santos Suehiro Arcos, Fabio Montoni, Wellington da Silva Santos, Hamida Macêdo Calacina, Ismael Feitosa Lima, Daniela Cajado-Carvalho, Emer Suavinho Ferro, Milton Yutaka Nishiyama-Jr, Leo Kei Iwai

**Affiliations:** 1 Laboratory of Applied Toxinology (LETA) and Center of Toxins, Immune-Response and Cell Signaling (CeTICS), Butantan Institute, São Paulo 05503-900, Brazil; carolkisaki@hotmail.com (C.Y.K.); stephanie.arcos@usp.br (S.S.S.A.); fabio.montoni@esib.butantan.gov.br (F.M.); wellington.silva@esib.butantan.gov.br (W.d.S.S.); hamidamacedo33@gmail.com (H.M.C.); ismael.lima@butantan.gov.br (I.F.L.); daniela.carvalho@butantan.gov.br (D.C.-C.); 2 Department of Pharmacology, Biomedical Sciences Institute (ICB), University of São Paulo (USP), São Paulo 05508-000, Brazil; eferro@usp.br

**Keywords:** mass spectrometry, proteome, snake venom, *Bothrops jararaca*, breast cancer

## Abstract

Cancer is characterized by the development of abnormal cells that divide in an uncontrolled way and may spread into other tissues where they may infiltrate and destroy normal body tissue. Several previous reports have described biochemical anti-tumorigenic properties of crude snake venom or its components, including their capability of inhibiting cell proliferation and promoting cell death. However, to the best of our knowledge, there is no work describing cancer cell proteomic changes following treatment with snake venoms. In this work we describe the quantitative changes in proteomics of MCF7 and MDA-MB-231 breast tumor cell lines following treatment with *Bothrops jararaca* snake venom, as well as the functional implications of the proteomic changes. Cell lines were treated with sub-toxic doses at either 0.63 μg/mL (low) or 2.5 μg/mL (high) of *B. jararaca* venom for 24 h, conditions that cause no cell death per se. Proteomics analysis was conducted on a nano-scale liquid chromatography coupled on-line with mass spectrometry (nLC-MS/MS). More than 1000 proteins were identified and evaluated from each cell line treated with either the low or high dose of the snake venom. Protein profiling upon venom treatment showed differential expression of several proteins related to cancer cell metabolism, immune response, and inflammation. Among the identified proteins we highlight histone H3, SNX3, HEL-S-156an, MTCH2, RPS, MCC2, IGF2BP1, and GSTM3. These data suggest that sub-toxic doses of *B. jararaca* venom have potential to modulate cancer-development related protein targets in cancer cells. This work illustrates a novel biochemical strategy to identify therapeutic targets against cancer cell growth and survival.

## 1. Introduction

Ophidian accidents constitute a serious public health problem in Brazil, with an average of 29,000 cases and 125 deaths reported every year (Brazilian Ministry of Health, 2019) [1]. Approximately 80% of ophidian accidents are caused by snakes of the Viperidae family, more specifically of the *Bothrops* genus [2]. Among them, about 25% lead to death or sequels capable of generating temporary or permanent incapacity for work and customary activities. Venom from the *Bothrops jararaca* (*B. jararaca*) snake is a complex mixture composed of proteins, peptides, amino acids, nucleotides, lipids, and carbohydrates that present a range of different actions when they are isolated or together [3,4,5,6,7], leading to hemotoxic, cardiotoxic, cytotoxic, or neurotoxic effects [8,9]. Several previous reports have defined the proteomics composition of *Bothrops* venoms [5,10,11,12,13,14,15,16]. These studies have shown that *Bothrops* venoms are composed of various classes of toxin, including metalloproteinases, serine proteinases, phospholipases A2, and C-type lectins, the most abundant components participating in the local and systemic envenomation effects.

The venom of *B. jararaca* engenders three main activities: proteolytic, coagulant, and hemolytic. The proteolytic activity causes degradation of extracellular matrix proteins, plasma, and cell surface [17], which represents an important factor for the clinical characterization of a bothropic accident [2,18]. In addition, venom can cause local tissue lesion, myonecrosis, edema, cardiovascular alterations, hypovolemic shock, coagulation alteration and renal alterations, resulting from the combined action of the enzymatic and toxic activity of the venom [19]. About 90 to 95% of the dry weight of the *B. jararaca* venom is composed of a complex mixture of proteins, mainly metalloproteinases, serine proteinases, phospholipases (PLA2), and L-amino acid oxidases. The metalloproteinases comprise most of the venom composition [20,21]. They are proteolytic enzymes associated with fibrinolysis and coagulation, and they are involved in cell migration and tissue repair, besides being related to pathological effects such as cancer [22,23]. In terms of therapeutic interventions, protease inhibitors have been shown to inhibit homeostasis and thrombosis by acting on the coagulation cascade [24]. The third major component of the venom, PLA2, is an enzyme capable of hydrolyzing the ester bonds at the sn2 position of glycerolphospholipids, releasing arachidonic acid, important for the biosynthesis of many mediators involved in inflammation, such as prostaglandins, thromboxanes, and leukotrienes [25]. Finally, the L-amino acid oxidases (LAAOs), which make up about 1 to 9% of the venom composition [26], are flavoenzymes belonging to the class of oxidoreductases, which produce alpha-keto acid, hydrogen peroxide, and ammonia [27,28]. However, when there is a high production of hydrogen peroxide, it has been found that L-amino acid oxidases can induce apoptosis in mammalian endothelial cells [29].

Snake venom constituents have been isolated and studied for their therapeutic potential in the treatment of various diseases. One example is Eptifibatide, marketed as Integrilin, derived from the *Echis carinatus* snake venom and produced by Millennium Pharmaceuticals and Schering-Plow. It is used as an antiplatelet drug [30]. Another example is the angiotensin I converting enzyme inhibitor Captopril produced by Bristol-Myers Squibb whose active component was derived from *B. jararaca* venom. It is used for the treatment against hypertension and renal insufficiency [31,32]. In addition to the potential use of the derivatives of snake venom toxins in the treatment of non-malignant diseases [33], several studies have described anti-tumorigenic characteristics of snake venom, stating that snake venom may be capable of inhibiting cell proliferation and promoting cell death by different means: inducing apoptosis in cancer cells by increasing the influx of Ca^2+^, inducing the release of cytochrome C, decreasing or increasing the expression of proteins that control the cell cycle, and causing damage to cell membranes [26,34,35,36]. With the goal of searching novel therapy against cancer, studies have characterized the proteins, peptides or enzymes derived from snake venom to identify components that are capable of interfering with the transport of substances or signal transduction across the membrane or disrupting the cell membrane [35,37].

With the rapid advances of nano-scale liquid chromatography (nLC) and mass spectrometry (MS) technologies in the last two decades, nLC-MS/MS-based proteomics analysis has been widely applied as a powerful tool for biomarker discovery to improve cancer therapy [38,39]. A number of studies have characterized the biochemical and physiological action of venom or isolated venom derivatives on cell lines or tissues [40,41,42,43]. In addition, several works have shown proteomic changes of cancer cell lines upon drug treatment that suggest molecular mechanisms of drug action, including diverse effects on proteasome regulation, metabolic processes, and oxidative stress [44,45].

However, to the best of our knowledge, there has been no report that describes the effects of *B. jararaca* snake venom treatment on breast cancer-related cell proteome. In the present study, nLC-MS/MS was used to characterize the effects of sub-toxic doses of *B. jararaca* snake venom on two different breast cancer cell lines MCF7 and MDA-MB-231. MCF7 and MDA-MB-231 are non-metastatic and metastatic tumor cell lines, respectively. They are characterized by a high degree of glycolytic efficiency that promotes the interaction between the tumor cell and the extracellular matrix [46]. Although both cell lines are from breast origin, they are molecularly distinct. MCF7 are estrogen and progesterone receptors positive and HER2 negative, while MDA-MB-231 are triple negative (estrogen receptor, progesterone receptor, and HER2 negatives) and prone to cytotoxic agents because of their impaired DNA repairing capability which is in part due to mutation in the p53 gene [47,48]. Proteomic changes observed herein upon treatment with *B. jararaca* snake venom in these cell lines highlight proteins and cell pathways that could be targeted in cancer therapy.

## 2. Results

### 2.1. The Cytotoxicity of B. jararaca Snake Venom in MCF7 and MDA-MB-231 Cells

**Figure 1:**
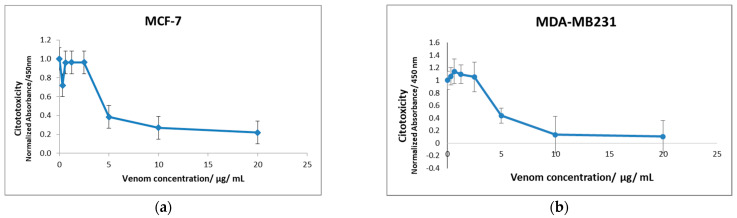
Cytotoxicity assay of (**a**) MCF7 and (**b**) MDA-MB-231 cell lines treated with *B. jararaca* snake venom ranging from 0 to 20 μg/mL for 24 h. Experiment was performed using the WST-1 reagent kit.

The *B. jararaca* venom cytotoxicity assay on MCF7 and MDA-MB-231 cell lines was monitored using the WST-1 reagent. This analysis showed that although cell viability was similar between both MCF7 and MDA-MB-231 cell lines, they had different venom resistance profiles where MDA-MB-231 cells showed to be more resistant to the venom when compared to the MCF7 cells. Although both cell lines started to die at doses higher than 2.5 μg/mL, at the 5.0 μg/mL of venom, only about half of the MDA-MB-231 cells have died while most of the MCF7 have died at this venom concentration (Figure 1). Lethal concentration 50 (LC50) was determined as 4.50 μg/mL for MCF7 and 4.76 μg/mL for MDA-MB-231 cell line. Interestingly, the treatment of cells with the low dose at 0.5 μg/mL of venom also killed more MCF7 cells compared to the MDA-MB-231 cells when compared to the venom doses of 0.63 μg/mL and 1.25 μg/mL (Figure 1).

### 2.2. Optical Microscopy Analysis of MCF7 and MDA-MB-231 Cells under B. jararaca Venom Treatment

Optical microscopy analysis at 10× magnification of MCF7 and MDA-MB-231 cell lines treated with concentrations higher than 2.5 µg/mL of *B. jararaca* snake venom showed cellular morphological changes such as cell shrinkage and cell birefringence change (Figure S1). At the 20 µg/mL of venom treatment all MCF7 cells detached from the plate, whereas the MDA-MB-231 cell line continued to show morphological death-like changes, but the cells did not detach from the plate (Figure S1).

Based on the cytotoxicity assays and visualization of cell morphology changes through the microscope images, two working concentrations, representing a low dose of 0.63 µg/mL and a high sub-toxic dose of 2.5 µg/mL of venom, were selected for further proteomics analysis.

### 2.3. Mass Spectrometry-Based Proteomics of MCF7 and MDA-MB-231 Cells Treated with B. jararaca Venom

**Figure 2:**
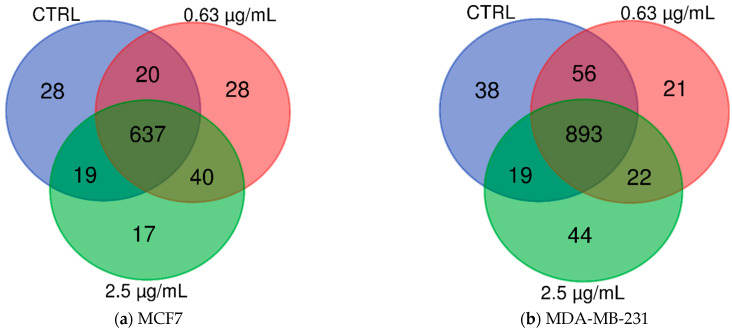
Diagrammatic representation of the comparative analysis of identified proteins under the three conditions: no venom control group, low venom dose at 0.63 µg/mL and high venom sub-toxic dose of 2.5 µg/mL of *B. jararaca* venom. (**a**) MCF7 and (**b**) MDA-MB-231.

Protein identification was performed analyzing the raw data using the MaxQuant software against the *Homo sapiens* database downloaded from Uniprot. Analysis of the MCF7 cell line treated with different venom concentrations allowed the identification of 789 proteins from which 704 proteins were identified in the control group (non-venom treatment), 725 proteins were identified in cells treated with 0.63 µg/mL of venom, and 713 proteins were identified in the cells treated with 2.5 µg/mL of venom. Comparative analysis of the identified proteins showed that among all of the 789 proteins identified, 637 proteins were in common to all conditions, 657 proteins were in common between the control and the 0.63 µg/mL venom treatment, 656 proteins were in common between control and the 2.5 µg/mL venom treatment, and 677 proteins were in common between the 0.63 µg/mL and 2.5 µg/mL venom treatments (Figure 2a, Table S1a). In addition, we also identified exclusive proteins in each condition: 28 proteins in the control group, 28 proteins in 0.63 µg/mL venom treatment, and 17 proteins exclusive in the 2.5 µg/mL venom treatment (Figure 2a, Table S1a).

In the MDA-MB-231 cell line, mass spectrometry-based proteomics analysis allowed us to identify a total of 1093 proteins from which 1006 proteins in the control group, 992 proteins in the cells treated with 0.63 µg/mL of venom, and 978 proteins in the cells treated with 2.5 µg/mL of venom. Comparative analysis of the identified proteins showed 893 proteins identified in common to all conditions, 949 proteins in common between the control group and 0.63 µg/mL venom treatment, 912 proteins were in common between control group and 2.5 µg/mL venom treatment, and 915 proteins were in common between the 0.63 µg/mL and 2.5 µg/mL venom treatments (Figure 2a, Table S1a). We also observed 38 exclusive proteins in the control group, 21 proteins in 0.63 µg/mL venom treatment, and 44 proteins exclusive in the 2.5 µg/mL venom treatment (Figure 2b, Table S1b).

### 2.4. Semi-Quantitative Proteomics Analysis: MCF7 and MDA-MB-231 Cell Line Protein Abundance Variation

In general, we observed a higher number of proteins whose abundance had changed more than 1.5× or less than 0.67× in MCF7 cell lines when compared to the MDA-MB-231 cell lines. The semi-quantitative analysis of the MCF7 cell line (Table S2a,) treated with 2.5 µg/mL venom allowed us to identify 137 proteins, whose abundance changed over 1.5× (fold change FC ≥ 1.5, marked in light red) when compared to the control group, from which 55 proteins presented FC ≥ 2.0 (marked in red). We highlight 12 highly abundant proteins with FC ≥ 3.0, marked in dark red: Sorting nexin-3 (SNX3), Purine nucleoside phosphorylase (HEL-S-156an), Peroxisome proliferator activated receptor interacting complex protein (PRIC295), Small nuclear ribonucleoprotein component (SNRP116), Eukaryotic translation initiation factor 4B (EIF4B), Methylcrotonoyl-CoA carboxylase beta chain (MCCC2), 26S proteasome non-ATPase regulatory subunit 5 (PSMD5), Heterogeneous nuclear ribonucleoprotein R (HNRNPR), Full-length cDNA clone CS0DJ015YJ12 of T cells (PSME2), Isoleucyl-tRNA synthetase (IARS), Large proline-rich protein BAG6 (BAG6), and Glutathione S-transferase (GSTM3). In addition, we identified 23 proteins with FC ≤ 0.67 (marked in light green) from which five proteins presented FC ≤ 0.5 (marked in dark green): Histone H4 (HIST1H4J), ATP synthase subunit d, mitochondrial (ATP5PD), Voltage-dependent anion-selective channel protein 2 (VDAC2), 4a-hydroxytetrahydrobiopterin dehydratase (PCBD), and Histone H3 (H3F3B). At the low 0.63 µg/mL venom treatment, we identified 25 proteins with FC ≥ 1.5 (marked in light red) from which only two proteins presented FC ≥ 2.0 (marked in red): Anterior gradient 2 homolog (AGR2) and Leucine-rich PPR-motif containing protein (LRPPRC); and 19 proteins with FC ≤ 0.67 (marked in light green) from which two proteins with FC ≤ 0.5 (marked in dark green): PCBD and 40S ribosomal protein S29 (RPS29). The description of the highlighted proteins is shown in Table 1.

Semi-quantitative proteomic analysis of the MDA-MB-231 cell line (Table S2b) treated with 2.5 µg/mL allowed the identification of 34 proteins whose abundance changed (FC) ≥1.5 over the control (marked in light red), nine proteins with FC ≥ 2 (marked in red) from which we highlight three proteins with FC ≥ 3 (marked in dark red): Histone H3.2 (H3C15/HIST2H3), 14 kDa phosphohistidine phosphatase (HEL-S-132P), and Mitochondrial carrier homolog 2 (MTCH2). Moreover, 41 proteins presented FC ≤ 0.67 (light green) from which we highlight four proteins with FC ≤ 0.5 (marked in dark green): DnaJ homolog subfamily A member 1 (DNAJA1), Insulin-like growth factor 2 mRNA-binding protein 1 (IGF2BP1), Cysteine-rich angiogenic inducer 61 (CYR61), and Thrombospondin-1 (THBS1). At the lower 0.63 µg/mL venom treatment, 16 proteins presented FC ≥ 1.5 (marked in light red) from which we highlight H3C15/HIST2H3 with FC = 3.8 and MTCH2 with FC = 2.3 (marked in red); and 28 proteins with FC ≤ 0.67 (marked in light green) from which 12 proteins with FC ≤ 0.5 (marked in dark green): 60S ribosomal protein L37 (RPL37), D-3-phosphoglycerate dehydrogenase (HEL-S-113), ATPase inhibitor, mitochondrial (ATP5IF1), Non-histone chromosomal protein HMG-14 (HMGN1), RCC2 protein (RCC2), Serine/threonine-protein phosphatase PP1-gamma catalytic subunit (PPP1CC), D-dopachrome decarboxylase (DDT), Ran GTPase-activating protein 1 (RANGAP1), dCTP pyrophosphatase 1 (DCTPP1), IGF2BP1, THBS1, and CYR61 (Table 1).

### 2.5. Hierarchical Clustering Analysis

**Figure 3:**
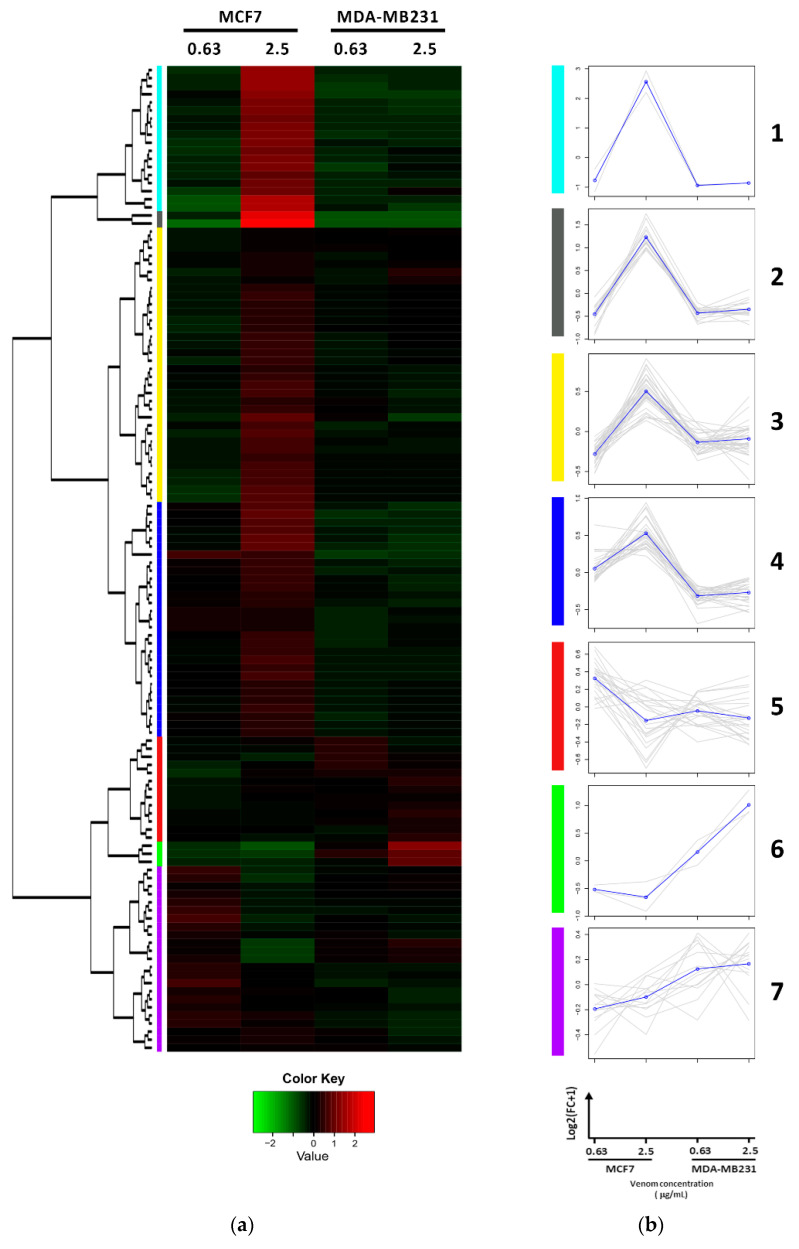
Hierarchical clustering of differentially expressed proteins detected in both MCF7 and MDA-MB-231 cells treated with low (0.63 µg/mL) and high (2.5 µg/mL) *B. jararaca* venom for 24 h. (**a**) Heatmap representation of the hierarchical clustering of proteins detected in both cell lines with quantification in at least two replicates showing the changes in protein abundance. The protein fold change is log2 transformed and normalized with mean-centering scale. (**b**) Protein Clusters extracted from the hierarchical clustering. X axis: Cell types treated with different *B. jararaca* venom concentrations (MCF7 0.63 μg/mL; MCF7 2.5 μg/mL, MDA-MB-231 0.63 μg/mL, MDA-MB-231 2.5 μg/mL); Y axis: mean-centered log2 Fold Change. Grey lines: individual proteins; Black line: average expression values per cluster.

Hierarchical clustering analysis of differentially expressed proteins with FC ≥ 1.5 identified in both MCF7 and MDA-MB-231 cell lines characterized those proteins in seven major clusters (Figure 3, Table S3). Clusters 1–4 had proteins with fold change (FC) increased at 2.5 µg/mL of venom treatment in MCF7 cells and presented no change in MDA-MB-231 cell lines. Proteins in these clusters include HEL-S-156 and PRIC295 in cluster 1 and PSMD5 and PSME2 in cluster 2. Cluster 5 identified proteins that decreased in FC at 2.5 µg/mL of venom treatment in both cell lines including HIST1H4J, VDAC1, and VDAC2. Cluster 6 identified proteins that increased FC at 2.5 µg/mL of venom treatment in MDA-MB-231 cell line including H3F3B, LAP3, and KRT1, and cluster 7 identified proteins that did not change when cells were treated with low and high venom treatment, but they presented higher FC change in MDA-MB-231 when compared to MCF7 cell line such as RPS29 (Table 1).

### 2.6. Principal Component Analysis

**Figure 4:**
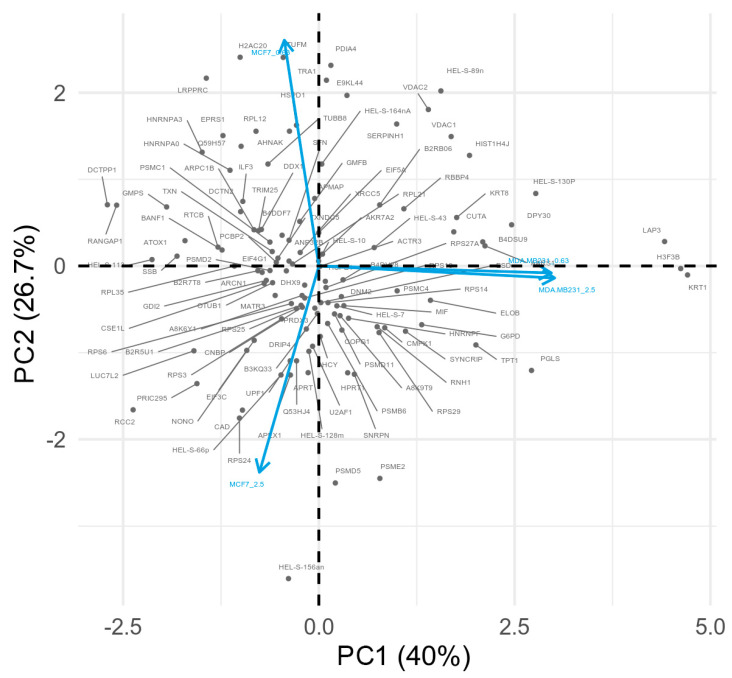
Comparison of protein log2 Fold Change profiles across treated cell lines. Principal component analysis in a 2D graph represented by the first two components PC1 and PC2 explains 67.7% of the protein variability among the different conditions. Vectors that are closer are highly correlated. Vectors representing the conditions which are orthogonal or well-spaced in terms of the observed proteome indicate that those proteins can be closely related to each specific cell line condition.

PCA was applied to the differentially expressed proteins identified from both MCF7 and MDA-MB-231 cell lines based on the log2 FC of cells treated with low and high *B. jararaca* venom compared to the PBS treatment control group (Figure 4). The projection into the component space shows a distinct coordinated activity of proteins between the cell lines conditions. Orthogonal vectors show highly positive correlation between both venom concentrations in MDA-MB-231 cell line which may represent similar cell line responses to the different venom concentrations, but they present a highly negative correlation to both venom concentrations in MCF7 and respective set of expressed proteins. In the MCF7 cell line, however, we observe a negative correlation between the 0.63 μg/mL and 2.5 μg/mL venom treatment, indicating a differential response upon low and high dose venom treatment. PCA also shows differential correlation between both cell lines where the first two components showed 40% variation in the PC1 and 26.7% variation in the PC2 between the MCF7 and MDA-MB-231 cell lines. In addition, we observed clusters of proteins positively correlating with both low and high venom treatment in MDA-MB-231 cell line such as LAP3, H3F3B, and KRT1. On the other hand, we also observed proteins such as H2AC20 and TUFM correlating with low venom treatment, and proteins such as HEL-S-156an correlating with high venom treatment on MCF7 cell line (Table 1).

### 2.7. Gene Ontology Functional Analysis 

The most enriched protein families and functional categories were analyzed based on highly abundant proteins with FC ≥ 1.5 for each cell line and treatment at low and high *B. jararaca venom* conditions (Figures S2–S5). The functional ontology classification analysis of these sets of proteins showed that both MCF7 and MDA-MB-231 venom-treated cell lines showed similar enriched categories. In addition, the most prominent enrichment was identified for treated cells with the sub-toxic dose of 2.5 µg/mL of venom. The molecular function enrichment analysis in both the MCF7 and MDA-MB-231 cell lineages showed an enrichment of proteins related to binding, structural molecule activity, and catalytic activity. In addition, the MCF7 cells had enriched, albeit in a lower amount, proteins related to function and transcriptional regulatory activity and carrier activity (Figure S2). The functional classification analysis related to biological processes showed enriched proteins related to the metabolic process and the cellular component organization or biogenesis. Moreover, the analysis of proteins identified in the MCF7 cell line presented proteins related to the cellular process, localization, biological regulation, stimulus response, developmental process, multicellular organismal process, and the immune system process (Figure S3). The analysis of protein distribution by cellular components showed an enrichment related to the “cell”, protein complex, and organelle (Figure S4), and the enrichment analysis of protein family classification showed an enrichment of cytoskeleton proteins, ligase, nucleic acid binding, signaling molecule, modulating enzyme, calcium binding protein, and hydrase (Figure S5).

### 2.8. Protein–Protein Interaction Analysis

STRING protein–protein interaction network analysis tool was used to evaluate protein–protein interactions identified among proteins with FC ≥ 1.5 from each cell line treated with *B. jararaca* venom. According to Doncheva and colleagues [114], STRING indicates interactions according to co-expression analyzes and evolutionary signals in all genomes based on data described in the literature between genes or proteins using functional classification systems such as Gene Ontology, KEGG (Kyoto Encyclopedia of Genes and Genomes), and Reactome.

**Figure 5:**
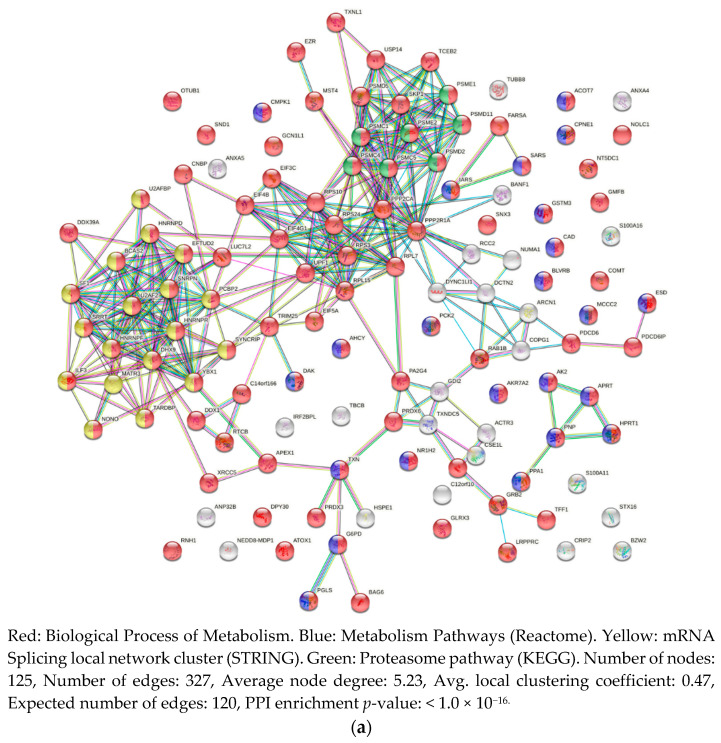

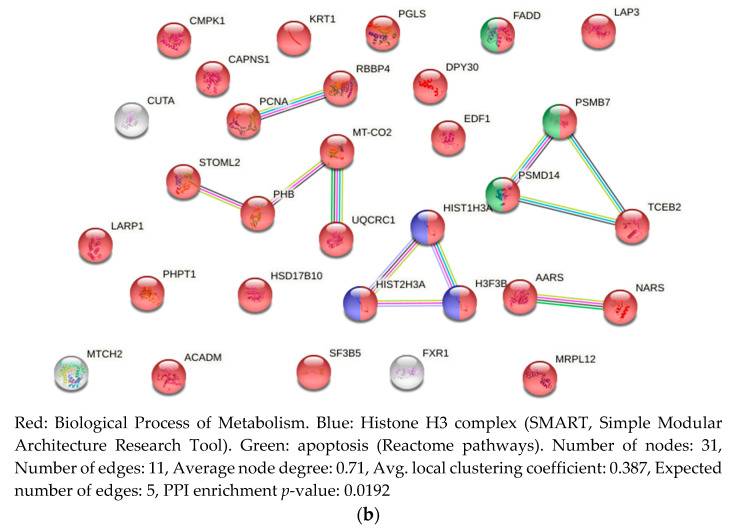
Protein–protein interaction of proteins identified in (**a**) MCF7 and (**b**) MDA-MB-231 cell lines presenting FC ≥ 1.5 at 2.5 g/mL *B. jararaca* venom treatment.

Analysis of protein–protein interactions (PPIs) of proteins whose abundance increased more than 1.5× in the MCF7 cell line treated with 2.5 μg/mL of venom showed a high interconnection of proteins related to metabolic process (in red) and metabolism pathways (in blue) (Figure 5a). Interestingly, among the proteins identified in the MCF7, we observed clusters of highly connected proteins related to proteasome pathway (in green) and mRNA splicing (in yellow). We highlight the proteins PSMD2 and PSMD11 (26S proteasome non-ATPase regulatory subunit 2 and 11, respectively), PSME1 and PSME2 (Proteasome activator complex subunit 1 and 2, respectively), PSMC1, PSMC4, and PSMC5 (26S proteasome regulatory subunit 4, 6B, and 8, respectively), TXNL (Thioredoxin-like protein 1), and USP14 (Ubiquitin carboxyl-terminal hydrolase 14), which are all members of the proteasome pathway (in green) and proteasome complex, and, together with MST4 (Serine/threonine-protein kinase 26) and PSMD5 (26S proteasome non-ATPase regulatory subunit 5) they are all related to apoptosis (Figure 5a). PPI analysis of proteins identified in the MDA-MB-231 in the same conditions showed less interaction among the proteins identified with FC ≥ 1.5 (Figure 5b).

PPI analysis of the MCF7 cell lines with FC ≥ 1.5 treated with 0.63 μg/mL and FC ≤ 0.67 when cells were treated with 0.63 μg/mL and 2.5 μg/mL of venom are shown in Figure S6. Additionally, PPI analysis of proteins identified in the MDA-MB-231 at 0.63 μg/mL and 2.5 μg/mL venom treatment presenting FC ≤ 0.67 and FC ≥ 2.5 are shown in Figure S7.

### 2.9. Exclusive Proteins

We further analyzed proteins exclusively identified in one or two of the three conditions in both MCF7 cell line (Table S4a, Figure S8) and MDA-MB-231 cell line (Table S4b, Figure S8). Proteins identified only on PBS treated cells (control group), suggesting that the expression of the proteins was inhibited with the addition of venom, allowed us to identify 28 proteins in the MCF7 cell line from which we highlight proteins: eukaryotic peptide chain release factor subunit 1 (ETF1), serine/arginine repetitive matrix protein 2 (SRRM2), PHD finger-like domain containing protein 5A (PHF5A), and lamina-associated polypeptide 2 (TMPO). The analysis of the MDA-MB-231 cell line allowed us to identify 41 proteins exclusively expressed at the control group from which we highlight Plasminogen activator inhibitor 1 (SERPINE1), MHC class I antigen (HLA-C), Transcription factor BTF3 (BTF3L4), Cytochrome c oxidase subunit 6C (COX6C), Myb-binding protein 1A (MYBBP1A), and MYCBP protein (MYCBP) (Figure S8a, Table S4).

Of the proteins identified exclusively when cells were treated with 0.63 μg/mL of venom there were 28 proteins from MCF7 cell line, from which we highlight Cytochrome b-c1 complex subunit 1, mitochondrial (UQCRC1), Latexin (LXN), Vesicle-associated membrane protein-associated protein B/C (VAPB), Cysteine and glycine-rich protein 1 (CSRP1), and DDB1- and CUL4-associated factor 7 (DCAF7). From MDA-MB-231 cell line, 20 proteins were identified from which we highlight Eukaryotic translation initiation factor 2 subunit 2 (EIF2S2), low molecular weight phosphotyrosine protein phosphatase (ACP1), Transcription factor A, mitochondrial (TFAM), and Rae1 protein homolog (RAE1) (Table 1 and Table S4, Figure S8b).

Proteins identified exclusively at the high dose treatment of 2.5 μg/mL of venom included 17 proteins detected in the MCF7 cell line, from which we highlight 26S proteasome non-ATPase regulatory subunit 6 (PSMD6) and 60S ribosomal protein L36 (RPL36). Similarly, we identified 44 proteins in MDA-MB-231 cell line from which we highlight protein Ubiquitin carboxyl-terminal hydrolase (HEL-117) (Figure S8c, Table S4).

We also identified proteins that were expressed exclusively when cells were treated with low and high doses of *B. jararaca* venom. From the MCF7 cell line, we identified 40 proteins from which we highlight: Serine/threonine-protein kinase ATR (ATR) and Jupiter microtubule-associated homolog 1 (JPT1). From the MDA-MB-231 cell line, we identified 22 proteins from which we highlight Angiomotin (AMOT) and Small nuclear ribonucleoprotein G (SNRPG) (Table 1, Tables S3 and S4, Figure S8d). 

Among the proteins that were identified in the control group and at the 2.5 μg/mL venom treatment, we highlight: Small nuclear ribonucleoprotein Sm D1 (SNRPD1) in MCF7, and Epididymis secretory protein Li 71 (Hel-S-71) and Isoform Far upstream element-binding protein 3 (FUBP3) in MDA-MB-231 cell line (Figure S8e, Table S4). Of proteins that were identified in the control group and low 0.63 μg/mL dose of venom, we highlight Serine/arginine-rich splicing factor 10 (SRSF10) in MCF7 cell line, and Histone H2B type 2-E (HIST2H2BE), 60S ribosomal protein L34 and L37a (RPL34, RPL37A) in MDA-MB-231 cell line (Figure S8f, Tables S3 and S4).

## 3. Discussion

The present study successfully identified cancer-related proteins that undergo significant changes upon *B. jararaca* venom treatment of MCF7 and MDA-MB-231 cells, including SNX3, HEL-S-156an, UQCRC1, RPL36, and ATR identified in MCF7 cell line and H3C15/HIST2H3, HEL-S-132P, MTCH2, TFAM, KCTD12, RPL34, and RPL37A identified in MDA-MB-231 cell line, and histone H3F3B and LAP3 in both cell lines.

With the discovery of rattlesnake venom’s antitumor activity in 1931 by Essex and Priestley [115] and later the angiotensin-converting enzyme inhibitor drug Captopril in 1981, developed originally from *B. jararaca* snake venom [116,117,118], several groups have been focusing on to the therapeutic potentials of bioactive compounds in snake venoms by isolating and characterizing components of the venom and analyzing their pharmacological potential that may lead to the development of more efficacious drugs [119,120].

Snake venoms have a complex mixture of proteins that can account for 95% of the total dried weight. Metalloproteases, serine proteases, LAAOs, and PLA2s are some of the most abundant proteins present in bothropic venoms, and the toxic action of these proteins are responsible for myotoxic effects, disruption in the coagulation cascade through thrombin-like proteins (serine proteases), imbalance in the blood homeostasis by the action of some metalloproteinases, apoptosis induction, and changes in the protein expression in oxidative stress and energy metabolism by the increase of Ca^2+^ influx. Several studies have revealed that these enzymes are critical regulators of cancer pathologies in several types of cancers including breast cancer [121,122,123,124,125,126,127]. Moreover, it has been previously shown that *B. jararaca* venom may have an important antitumor effect on Ehrilich ascites tumor cells in vivo and in vitro [36].

Aiming at the drug development, several groups have used proteomics approach to observe up- or down-regulated effects of proteins resulting from disease activity or side effects of the treatments [44,45,128,129,130,131]. Moreover, the quest for the identification of biological markers or biomarkers using venom that may help in the early detection of pathologies such as cancer, and the quest for products able to evaluate the metastatic potential and propose new forms of treatment to tumors, remains an important goal of research groups and pharmaceutical companies.

Proteomics studies using tumor cell lines has been performed to systematically characterize protein complexes that may be important in disease or drug development, comparing the protein levels of cells in normal versus pathological situations [132]. Studies have shown that venoms from several snakes such as *Calloselasma rhodostoma*, *Macrovipera lebetina* and *Bothrops mattogrossensis* significantly inhibit cell viability, either with crude or fractionated venoms and in different tumor cell lines such as LS174T (colorectal adenocarcinoma), HCT116 (colorectal carcinoma), HT29 (colorectal adenocarcinoma), HEL92.1.7 (erythroleukemia), and SK-BR-3 (breast adenocarcinoma) cell lines [133,134].

To evaluate the cytotoxicity of *B. jararaca* venom, we tested two different invasive breast cancer cell lines with different phenotypic and genotypic differences, MCF7 and MDA-MB-231 cell lines. They were treated with different venom concentrations ranging from 0.32 µg/mL to 20 µg/mL. When treated with over 10 µg/mL of venom both MCF7 and MDA-MB-231 cell lines showed rounded and irregular morphologies and the MCF7 cell line detached from the plate. These results are similar to the results observed by Bernardes-Oliveira et al. (2016) when testing 50 µg/mL of venom from *B. jararaca* and *Bothrops erythromelas* for 48 h on SiHa HPV-16 and HeLa cells, both derived from cervical tumors. They observed that the cells became more rounded, with gradual size reduction and detachment from the cell monolayer [135]. In addition to the morphological analyses, we evaluated cell viability through mitochondrial cell activity using WST-1 assay for cell proliferation and viability. We observed that cell death of both MCF7 and MDA-MB-231 cell lines started with 2.5 µg/mL of *B. jararaca* venom treatment. Based on both morphological and cell viability analyses, we selected the concentrations of 0.63 µg/mL (low sub-toxic dose) and 2.5 µg/mL (high sub-toxic dose) for further proteomic analyses.

Semi-quantitative proteomics analysis of the MDA-MB-231 cell line treated with the low 0.63 μg/mL and the sub-lethal dose of 2.5 μg/mL of *B. jararaca* venom presented less proteins with FC ≥ 1.5 when compared to the MCF7 cell line. We identified 137 proteins with FC ≥ 1.5 at 2.5 μg/mL venom treatment in MCF7 while 34 proteins in MDA-MB-231 at 2.5 μg/mL venom treatment. At the low 0.63 μg/mL venom treatment, 25 proteins in MCF7 presented FC ≥ 1.5 and 16 proteins in MDA-MB-231. However, MDA-MB-231 showed more proteins with FC ≤ 0.67 compared to MCF7 cell lines. MDA-MB-231 showed 28 proteins presenting FC ≤ 0.67 at 0.63 μg/mL venom treatment and 19 proteins in MCF7; and 41 proteins with FC ≤ 0.5 at 2.5 μg/mL venom treatment in MDA-MB-231 cell lines compared to 23 proteins in MCF7 cell line. Among these proteins, our data showed that *B. jararaca* venom was able to modulate, up or down, the abundance of several proteins playing essential roles in tumorigenicity. For example, the venom treatment increased the abundance of SNX3, HEL-S-156, MCCC2, and GSTM3 in the MCF7 cell line, and H3C15/HIST2H3 and MTCH2 in the MDA-MB-231 cell line whose overexpression has been related to increase in tumorigenicity (Table 1). Furthermore, among the proteins that decreased the abundance presenting FC ≤ 0.67, we highlight PCBD, PSMD5, RPS29, H3F3B, VDAC1 and VDAC2, ATP5PD, and HIST1H4J in MCF7 cell line and IGF2BP1, THBS1, and CYR61 in MDA-MB-231 cell line. These proteins have also been described to be related to several mechanisms and pathways in the onset of different types of cancer (Table 1). Additionally, cytotoxicity analysis performed in our laboratory (data not shown) evaluated the effects of *B. jararaca* venom on non-tumor HEK293 and HUVEC cell lines, which showed relatively less sensitivity to cell-death induction by snake venoms compared to MCF7 and MDA-MB-231 cancer cells. These data suggest that cancer cells could be more sensitive to snake venom components than non-cancer cells in certain situations.

Proteomic analysis also revealed exclusive proteins, i.e., proteins that were only identified in one or two of the three conditions analyzed. Among these proteins, we highlight the UQCRC1, identified in the MCF7 cell line only when these cells were treated with 0.63 µg/mL venom. High expression of this protein was observed in 74% of cases of breast cancer and 34% of ovarian cancer [104] (Table 1). Among proteins identified exclusively in the MCF7 cell line we highlight ATR (serine/threonine-protein kinase ATR) which plays important roles for cell survival and is considered a major mediator of DNA response in human cells, preventing cells with damaged or incompletely replicated DNA from entering mitosis when cells are damaged by radiotherapy or chemotherapy during cancer treatment [53]. In the MDA-MB-231 cell line, we highlight AMOT (angiomotin) protein identified only when cells were treated with either low or high concentration venom treatment and is known to play a critical role in angiogenesis, proliferation, and migration of cancer cells and is also known to promote proliferation and invasion of several types of tumor cells, including breast, prostate, colon, cervical, and liver cancer (Table 1).

Semi-quantitative proteomics analysis of proteins with FC ≥ 1.5 after addition of venom in both MCF7 and MDA-MB-231 cell lines also identified enrichment of proteins related to protein metabolism pathways as well as cellular stress response pathway. The protein metabolism pathway ranges from protein synthesis to post-translational protein modification and degradation [136]. It is likely that the set of modifications undergone in the protein metabolism pathway undermine the tumorigenic activity of the cell line MCF7 and MDA-MB-231. Therefore, the activation of the apoptotic pathways using snake venom may be a potential treatment approach aiming at tumor degradation preferentially over the normal cells. The ribosome pathway enrichment was also observed in both cell lines. The lack of ribosomes in the cell impairs cell growth even under optimal conditions for it to happen [137]. Protein enrichment in ribosome pathway suggests increased protein synthesis, which may be related to damage repair mechanisms and cellular responses to external stimuli. Alterations in ribosomal pathways are related to cellular alterations and susceptibility to cancer. However, there is still no consensus if deregulation of ribosomes alteration is a cancer consequence or cause [138]. In addition, downregulation of cell maintenance-related proteins may also be related to tumorigenicity, thus opening the door for debate on the role of ribosomes in tumor cell lines [139].

The increase of terms involved in cellular stress response pathway, the metabolic pathway, the proteasome pathway, and the spliceosome pathway in MCF7 cell line suggests that the cells activated the metabolic pathways in response to stress, mRNA transcription and spliceosome activation, and ultimately activation of the proteasome. The proteasome is a cell apparatus that has several cellular functions, such as cell cycle regulation, differentiation, signal transduction pathway, antigen processing for immune responses, stress signaling, inflammatory responses and apoptosis, the latter being mediated by ubiquitination [140,141,142]. Since the inhibition of proteasome is related to cancer onset (i.e., p53 stability), proteolytic activity of the proteasome and its role in both biology and cancer treatment suggest an effective way to treat cancer [143,144].

In the MCF7 cell line treated with *B. jararaca* snake venom, a higher number of proteins changed in abundance when compared to the MDA-MB-231 cell line. Some of our observations showed that *B. jararaca* venom was able to up or down modulate the abundance of proteins that plays essential roles in tumorigenicity, conversely to what has been frequently described in the literature to decrease or increase cell tumorigenicity [113,145]. For example, the venom treatment was able to increase abundance of SNX3, HEL-S-156, MCCC2, and GSTM3 in the MCF7 cell line and H3C15/HIST2H3 and MTCH2 in the MDA-MB-231 cell line whose overexpression is related to increase in tumorigenicity. Although we cannot explain the mechanism on how the increase in abundance of these already overexpressed proteins is contributing to cell death of MCF7 and MDA-MB-231 cells, we have clues based on a recent published manuscript by Dias and colleagues (2019). The authors demonstrated that overloading the cell stress pathway in Y1 adrenocortical mouse tumor cell line under the effect of FGF-2 growth factor disrupted the cell homeostasis and sensitized the Y1 tumor cells to stress-oriented therapeutic inhibitors. Dias concludes that further stimulation of the same signaling pathways may further increase mobilization and dependence on stress response pathways in tumor cells, improving the efficacy and selectivity of therapeutic interventions [146]. This data suggests that *B. jararaca* snake venom or some of its content may be used to down-modulate already known overexpressed proteins in cancer or overexpress oncogenic proteins that lead cells to stress, by disrupting the tumor homeostasis leading cells to death. This is particularly interesting for cancer types that are more resistant to the chemotherapeutic agents, such as the MCF7 cells. In this case, *B. jararaca* snake venom would work as an adjuvant treatment of cancer, making cells more sensitive to the cytotoxic agents.

Moreover, due to the complexity of *B. jararaca* venom and the complex response of different cell lines, this snake venom is also a promising candidate in the prognostic aid of different tumors, assisting in the assessment of tumor level, as highlighted for the several overexpressed proteins. For example, targeted-knockdown of some of these proteins may result in tumor growth inhibition. In addition, *B. jararaca* venom has induced overexpression of proteins related to pathways that impairs antitumor activity such as the protein metabolism pathways, proteasome, and cellular stress response pathways.

It is possible that many responses observed here were due to specific or multiple venom components. Nevertheless, the current approach of applying nLC-MS/MS proteomics to cancer cells treated with crude snake venom is a promising strategy for the identification of proteins with potential application in cancer cell therapy. Overall, this study was able to identify several cancer-related proteins that undergo significant changes upon venom treatment of MCF7 and MDA-MB-231 cells. Future studies should address specific mechanisms by which the snake venom and some of its content may contribute to MCF7 and MDA-MB-231 cell death and survival.

## 4. Conclusions

Quantitative proteomic analysis of breast cancer cell lines allowed us to identify several proteins whose abundance (FC) increased more than 1.5×, and proteins that abundance decreased less than 0.67×, after 24 h treatment with *B. jararaca* at either low sub-toxic dose of 0.63 μg/mL or high sub-toxic dose of 2.5 μg/mL. Most of these proteins identified suggest that the treatment with the venom may activate mitochondrial apoptotic pathways leading cells to death. In addition, several of the identified proteins play important roles related to cell proliferation, invasion, metastasis, apoptosis, and stress response. Therefore, these data show that *B. jararaca* venom or some of its toxin or components can inhibit tumor cell proliferation and survival and can potentially be used to identify novel targets for cancer therapy.

## 5. Materials and Methods

### 5.1. Cell Culture and Maintenance

MCF7 and MDA-MB-231 breast cancer cell lines were previously acquired from ATCC (Manassas, VA, USA) and maintained at our laboratory cell bank. Cells were thawed and cultured at 37 °C in 5% CO_2_ in RPMI 1640 culture medium (Gibco, Life Technologies, Grand Island, NY, USA) supplemented with 10% inactivated fetal bovine serum (Cultilab, Campinas, Brazil) and 25 mg/mL ampicillin and 100 mg/mL streptomycin antibiotics. Cells were split when the confluence reached 80%.

### 5.2. Bothrops jararaca Venom

The venom of *B. jararaca* (lot 01/09-2) used in this study was a pool made from the extraction of 697 snakes collected at various Brazilian locations (in the states of São Paulo, Paraná, and Santa Catarina). Extracted venom were pooled and lyophilized at the department of herpetology at Butantan Institute under the coordination of Dr. Marisa Maria Teixeira da Rocha, and assigned for use and testing at the Laboratory of Applied Toxinology at Butantan Institute. The methods and use of venom in this work were approved by Butantan Institute Ethics Committee under the certification CEUAIB #9766150719 (2019).

### 5.3. Cell Viability Assay

The cytotoxicity assays were carried out by treating the cells with different concentrations of venom in the range from 0.1 μM to 20 μM. The cytotoxicity tests were performed using the colorimetric method of cell viability analysis using the WST-1 cell proliferation reagent kit (Roche, Mannheim, Germany) according to manufacturer’s instructions. Briefly, 10^4^ cells were plated in each well of a flat bottom 96-well microtiter plate. Cells were cultured at 37 °C in 5% CO_2_ and on the day prior to analysis (70–80% confluency), culture medium from each well was exchanged to 100 μL of medium in the absence or presence of venom in different concentrations. After 24 h, 10 μL of the WST-1 reagent previously dissolved into an Electro Coupling Solution (ECS) were added to each well and incubated for 4 h at 37 °C in 5% CO_2_. The absorbance at 450 nm of each well was measured using a microplate reader FlexStation 3 spectrophotometer (Molecular Devices, San Jose, CA, USA). The determination of the absorbance and the quantification of viable cells were calculated using SoftMax Pro (version 5.1, Molecular Devices) and Microsoft Excel software.

### 5.4. Cell Treatment with B. jararaca Venom

In a six-well plate, 2 × 10^5^ cells were plated and after the cells had reached a confluence of 70–80% (2–3 days), different concentrations of *B. jararaca* venom were added to the culture medium, starting from the cell cytotoxicity threshold up to a 1000-fold dilution for 24 h. For mass spectrometry analysis, cells were washed with ice-cold PBS and lysed with 1 mL ice-cold 8 M urea supplemented with cOmplete^TM^ protease and phosphatase inhibitors cocktail (Merck Millipore, Burlington, MA, USA). Fifty microliters of the cell lysate were separated for protein quantification using BCA (Bicinchoninic acid assay, Thermo Pierce, Walthan, MA, USA) and the lysate were stocked at −80 °C until further preparation of the sample for mass spectrometry analysis. Both cytotoxicity and proteomics experiments were performed in three biological replicates.

### 5.5. Sample Preparation for Proteomic Analysis

To the cell lysates, four microliters of 10 mM dithiothreitol (DTT) were added to reduce the disulfide bonds and incubated at 56 °C for 1 h. After incubation with DTT, 40 μL of iodoacetamide (IAA) was added for the alkylation of the cysteines for 1 h in the dark at room temperature. After incubation, 2.5 mL of ammonium acetate and 20 μL of trypsin (Sigma Aldrich, St. Louis, MO, USA) were added enough to have an enzyme:protein ratio of 1:50 and incubated at 37 °C overnight. The reaction was stopped by adding 30 μL of 100% acetic acid. Samples volume were reduced in a SpeedVac (Hetovac VR-1, Heto Lab Equipment, Allerød, Denmark) until the volume has lowered to 50 μL and were further desalted on in-house manufactured stop-and-go extraction tips (Stage-Tip) with three SDB-XC (styrene-divinylbenzene, Empore, 3M, Royersford, PA, USA) membranes as previously described [147]. The stage-tips were initially conditioned with 100% methanol followed by 0.1% formic acid. Samples were applied to the stage-tips and the peptides bound to the membranes were eluted with 50% acetonitrile, 0.1% formic acid. The eluate was lyophilized for further analysis in the mass spectrometer.

### 5.6. Mass Spectrometry Analysis

Dried samples were resuspended in 0.1% formic acid and analyzed on LTQ-Orbitrap Velos mass spectrometer (Thermo Scientific, Bremen, Germany) coupled to an EASY II nano liquid chromatographer (Thermo Scientific) using the shotgun approach. The spectrometer was equipped with a nanospray source connected to an in-house prepared analytical column (10 cm × ID 75 μm × OD 360 μm) packaged with 7 cm of 5 μm C18 resin (Jupiter, Phenomenex, Torrance, CA, USA). Precolumn (7 cm × ID 75 μm × OD 360 μm) was also prepared in-house packed with 5 cm of 10 μm C18 resin (Acqua, Phenomenex). The LC-MS/MS analyses were carried out by injecting 5 μg of the peptide extract and the peptides eluted from the column with a gradient of 5–40% in 100 min of solvent B (acetonitrile 90%, formic acid 0.1%) at a flow rate of 200 nL/min. The nanospray source was operated at 1.8 kV. The peptide mixture was analyzed by the acquisition of spectra in the full scan mode at a resolution of 30,000 for the determination of molecular masses (MS) of up to 10,000 Da. The 10 most intense peaks were automatically selected via data dependent acquisition (DDA) for the subsequent acquisition of spectra of the ions product to MS/MS for amino acid sequence determination at a resolution of 7500, a maximum injection time of 30 ms, a range of 200 to 2000 *m*/*z*, and a dynamic exclusion of 70 s. Protein identification was performed using Mascot (Matrix Science, version 2.4.0, Boston, MA, USA), MaxQuant (version 1.4.1.2, www.maxquant.net accessed on 1 October 2019), and Peaks Studio (version 10, Bioinformatic Solutions Inc, Toronto, Canada) against the *Homo sapiens* database downloaded from the Uniprot in March 2019 (www.uniprot.org accessed on 1 October 2019). As search parameters, oxidation of methionine was set as a variable modification and carbamidomethylation of cysteine was set as a fixed modification. Searches were performed with mass error tolerance of 10 ppm for MS and 0.3 Da for MS/MS, and trypsin was selected as the proteolytic enzyme used in the digestion of proteins, and up to two miscleavages were allowed. Mass spectrometry-based proteomic analysis of MCF7 and MDA-MB-231 cell lysates, treated or not treated with low or high doses of *B. jararaca* venom were performed in triplicate. All raw data files for these analyses were uploaded and are available at: http://massive.ucsd.edu/MSV000084138/ accessed on 5 July 2021.

### 5.7. Proteome Functional and Enrichment Analysis

The entire proteome was analyzed to classify protein profiles by biological processes, molecular function, cellular components, and cellular pathways using Gene Ontology (geneontology.org accessed on 1 October 2019) and PantherDB (pantherdb.org accessed on 1 October 2019). The proteins presenting different profiles in the venom conditions were also analyzed using KEGG (www.genome.jp/kegg accessed on 1 October 2019) canonical pathways to determine the protein group or pathways that have undergone changes with different concentrations of the venom treatment. The PANTHER Classification System (Protein ANalysis THrough Evolutionary Relationships) is a research tool that covers evolutionary and functional information on protein family genes and has been widely used to understand protein evolution and its functional classification [148,149]. We conducted the functional classification for the molecular function, cell component, biological process, and protein family classification profiles [150,151] of the different proteins in each cell line and treatment at low and high *B. jararaca* venom that presented FC ≥ 1.5 in order to identify an enrichment pattern of protein families and pathways affected by the venom in the cell lines.

### 5.8. Semi-Quantitative Proteomics Analysis

Semi-quantitative protein analysis was performed using the Label Free Quantification MaxLFQ algorithm from MaxQuant software with an FDR rate of ≤1% to compare relative abundance of proteins in each of the cell lines [152]. Data generated by MaxQuant were entered into the Perseus program to further perform statistical and bioinformatics analyzes [153]. Proteins identified in the contaminant database and the decoy database were removed. As a protein identification criterion, it was considered that only peptides identified with the posterior error probability (PEP) ≤ 0.01 in at least one biological replicate, the minimum identification of eight ions belonging to the b and y ion series in the MS/MS spectra, and the occurrence of at least one unique peptide. We considered the intensity values of the LFQ that are normalized by the Maxquant software based on the sum of the intensity of all peptides of all identified proteins. LFQ data was considered for calculation, when the intensity data were present in at least two out of three replicates. Protein abundance or fold change (FC) analysis was performed using Microsoft Excel software. In addition, proteins that had a zero value in two of the three conditions were analyzed separately. The breast cancer cell lines treated with the whole *B. jararaca* venom for two different doses were compared according to the differentially expressed proteins with FC ≥ 1.5 by hierarchical clustering using as variables the average log2 fold change for the three replicates for each treatment, normalized with mean-centering. The Clustering analysis was performed using R statistical software version 3.6.3 (http://www.R-project.org accessed on 1 October 2019). The set of protein dissimilarities were computed using the “Euclidean” distance with the function “dist” to the hierarchical clustering based on the package and function “hclust”. There was employed the agglomerative method with “ward.D2”. The fold change and the protein–protein interactions provided by information from the String database [154] was used to explore the biological interactions of proteins identified as differentially abundant between control and cells treated with *B. jararaca* venom using the *Homo sapiens* reference genome.

### 5.9. Principal Component Analysis

Principal component analysis (PCA) was applied to find which linear combinations of the differentially expressed proteins with ≥1.5 would explain most of the variability for the different cell lineage conditions. The PCA analysis was performed using the R-statistics packages FactoMineR (accessed on 1 November 2020) [155] and Factoextra (http://www.sthda.com/english/rpkgs/factoextra accessed on 1 November 2020) for graphical visualization.

## Figures and Tables

**Table 1:** Description of the highlighted proteins and their association with cancer.

Protein	Protein Name	Protein Description Related to Cancer	Cancer Type Association	References
AMOT	Angiomotin	Plays a central role in tight junction maintenance. Appears to regulate endothelial cell migration and tube formation. May also play a role in the assembly of endothelial cell-cell junctions. Plays a critical role in angiogenesis, proliferation and migration and invasion of cancer cells	BL, BR, CE, CL, CR, EN, HN, KD, LE, LI, LA, LS, OV, PR, ST	[49,50,51]
ATP5PD	ATP synthase peripheral stalk subunit D	Mitochondrial ATP synthase catalyzes ATP synthesis, utilizing an electrochemical gradient of protons across the inner membrane during oxidative phosphorylation. Linked to failure of therapy, disease progression, and poor survival in patients with cancer. High expression of ATP5PD has been observed in several types of cancer	BR, CE, CL, EN, GL, HN, LI, LU, LY, ME, OV, PA, PR, RE, SK, TE, TY	[52]
ATR	Serine/threonine-protein kinase	Plays important roles for cell survival and is considered a major mediator of DNA response in human cells, preventing cells with damaged or incompletely replicated DNA from entering mitosis when cells are damaged by radiotherapy or chemotherapy during cancer treatment	BL, BR, CE, CR, EN, GB, HN, KD, LU, LA, LS, OV, ST, TY	[53,54]
CYR61	Cysteine-rich heparin-binding protein 61	Plays an important role in cell proliferation, survival, chemotaxis, angiogenesis, adhesion, and migration of different types of cells. Participate in key different cellular events during vascular development, angiogenesis, wound healing and the development and progression of various types of cancers	BN, BR, CR, EN, GA, GB, GL, LI, LU, OV, PA, PR, ST, TE, UR	[55,56]
GSTM3	The glutathione S-transferase Mu 3	Part of the GSTs enzymes that have functions such as immunological system evasion and inhibition of apoptosis. Involved in prostaglandin and leukotriene synthesis and metabolization of both endogenous compounds and xenobiotics such as chemotherapeutic drugs, insecticides, carcinogens, and oxidative stress byproducts	BL, BR, CR, EN, LE, LA, LS, OV, PA, ST, TY, UR	[57,58]
H3F3B/H3C15	H3 histone family member 3B	Core component of nucleosome. Histones play a central role in transcription regulation, DNA repair, DNA replication and chromosomal stability and are related to different types of cancer. H3F3B mutation has been described to lead to some human cancers	BL, BN, BR, CE, CH, CR, EN, GB, HN, LU, OV, UR	[59,60,61]
HEL-S-156an PNP	Purine nucleoside phosphorylase	Catalyze the phosphorolysis of purine nucleosides. Mutations which result in nucleoside phosphorylase deficiency result in defective T-cell (cell-mediated) immunity but can also affect B-cell immunity and antibody responses. High expression of PNP has been observed in several types of cancer	BR, CL, CR, GA, GL, KD, LI, LU, LA, LY, ME, OV, PR, TY	[62,63,64]
HIST1H4J	Histone H4	Core component of nucleosome. Histones play a central role in transcription regulation, DNA repair, DNA replication and chromosomal stability. Post-translational alterations of histones have been shown to affect the activation and repression of oncogenes and tumor suppressor genes	BR, CE, CR, GL, HN, LI, LU, ME, OV, PA, PR, SK, ST, TE, TY	[65,66,67,68]
IGF2BP1	Insulin like growth factor 2 MRNA binding protein 1	RNA-binding factor that recruits target transcripts to cytoplasmic protein–RNA complexes (mRNPs). IGF2BP1 has an oncogenic role, characterized by changes in actin dynamics, migration, invasion, proliferation, and self-renewal. Play a role in resistance to drugs	BR, CE, CR, EN, GB, HN, LI, LU, LA, LS, ME, OV, PR, ST, TE, UR	[69,70,71]
KRT1	Keratin	May regulate the activity of kinases such as PKC and SRC via binding to integrin beta-1 (ITB1) and to the receptor of activated protein C kinase 1 (RACK1). High expression of KRT1 protein has been observed in several types of cancer and is correlated with advanced melanoma tumor stage and infiltration of immune cells	BR, CE, CR, EN, GB, HN, KD, LI, LS, LA, OV, SK, ST, UR	[72,73]
LAP3	Leucine aminopeptidase 3	Cytosolic metallopeptidase that catalyzes the removal of unsubstituted N-terminal hydrophobic amino acids from various peptides. Involved in the metabolism of glutathione and in the degradation of glutathione S-conjugates, which may play a role in the control of the cell redox status. Related to protein renewal. Have a potential for determining the prognosis for breast cancer	BL, BR, CR, EN, HN, KD, LI, LA, LS, PA, ST, TY	[74,75,76,77]
MCCC2	Methylcrotonoyl-CoA carboxylase beta chain, mitochondrial	Enzyme that catalyzes the conversion of 3-methylcrotonyl-CoA to 3-methylglutaconyl-CoA, a critical step for leucine and isovaleric acid catabolism. Overexpression of MCCC2 is associated with tumor stage, node, metastasis, lymph node metastasis and predicts unfavorable prognosis. Additionally, involved in the development and formation of some tumors, such as breast cancer	BL, BR, CR, EN, HN, KD, LI, LA, OV, PA, PR, ST, TY	[78]
MTCH2	Mitochondrial carrier homolog 2	Member of the SLC25 family of nuclear-encoded transporters that are localized in the inner mitochondrial membrane. Members of this superfamily are involved in many metabolic pathways and cell functions. Associated with metastasis and tumor cell survival. Indirect involvement in the expression of miR-135b mRNA, which is one of the proteins responsible for tumorigenicity	BL, BR, CR, EN, HN, KD, LI, LA, ME, OV, PA, PR, ST, TY	[79,80,81]
PCBD	4a-hydroxytetrahydrobiopterin dehydratase	Involved in tetrahydrobiopterin biosynthesis. Regulates various aspects of cell morphogenesis and differentiation as a cofactor for the homeobox transcription factor. Several types of cancer show expression or alteration in the homeobox genes. PCBD degradation increases cell survival and proliferation, and inhibits tumor cell differentiation	BR, CL, CR, EN, LE, LI, LU, OV, PA, PR, RE, SK	[82,83,84]
PRIC295	Peroxisome proliferator-activated receptor-a (PPARα)-interacting cofactor	Functions as a transcriptional coactivator for nuclear receptors. Enhances the activation of PPARα and PPARγ and plays a key role in lipid metabolism and energy combustion regulating the genes for fatty acid oxidation. Observed to be significantly enhanced in chemotherapy recurrence when compared to chemotherapy treatment in ovarian cancer patients	BL, BR, CR, EN, GB, HN, KD, LE, LA, LS, ME, OV, PA, PR, ST, TY	[85,86,87]
PSMD5	The 26S proteasome non-ATPase regulatory subunit 5	Acts as a chaperone during the assembly of the 26S proteasome. Expression reduced in several types of cancer including intestinal and colorectal tumors	BL, BR, CR, EN, HN, PR, ST, TY	[88]
PSME2	The proteasome activator complex subunit 2	Implicated in immunoproteasome assembly and required for efficient antigen processing. Member of the PSME family that regulates proteasome function. Elevated expression of PSME have also been associated with several types of cancer	BR, CR, EN, HN, LC, LA, LS, ME, PR, ST	[89,90,91]
RPS29	Ribosomal protein S29	Belongs to the universal ribosomal protein uS14 family. Related to have tumor suppressor activity for ras-transformed NIH3T3 cells. High expression RPS29 mRNA levels observed in adenomas	BR, CE, CR, EN, HN, KD, LA, OV, ST	[92,93]
SNRP116/EFTUD2	Small nuclear ribonucleoprotein component	Required for pre-mRNA splicing as a component of the spliceosome, including pre-catalytic, catalytic, and post-catalytic spliceosomal complexes. Knockout of EFTUD2 suppressed the development and tumor progression due to impaired activation of NF-kB signaling in macrophages	BL, BR, CR, EN, GB, HN, KD, LA, LS, ME, OV, PR, ST, TE, TY, UR	[94,95]
SNX3	The sorting nexin 3	Phosphoinositide-binding protein required for multivesicular body formation. Plays a role in protein transport between cellular compartments. The knockdown of SNX3 is associated with degradation of the EGF receptor which is related to resistance to chemotherapy and radiotherapy	BR, CR, EN, GL, HN, KD, LU, OV, RE, TY	[96,97]
THBS1	Thrombospondin 1	Adhesive glycoprotein that mediates cell-to-cell and cell-to-matrix interactions. Influences angiogenesis modulation by regulating adhesion, invasion, metastasis, migration, proliferation, and apoptosis and has been implicated in numerous types of cancers	BR, CR, EN, ES, HN, KD, LA, LS, LY, ME, OV, PA, PR, SK, ST, TE, TY	[98,99,100]
TUFM	Tu translation elongation factor, mitochondrial	Promotes the GTP-dependent binding of aminoacyl-tRNA to the A-site of ribosomes during protein biosynthesis. Plays important roles in the regulation of autophagy and innate immunity. TUFM is highly expressed in several types of cancers	BR, CR, EN, ES, GA, GS, HN, LI, LU, LA, ME, PA, PR, RE, SK, ST, TE	[101,102,103]
UQCRC1	The ubiquinol-cytochrome C reductase core protein 1	Component of the ubiquinol-cytochrome c reductase complex, which is part of the mitochondrial respiratory chain. High expression was observed in several types of cancer. Negative expression correlated significantly with clinical and pathological parameters including tumor stage, vascular invasion, and lymph node metastasis, suggesting that the reduction of this protein is associated with tumor progression	BR, CR, EN, GB, GL, HN, LI, LA, LY, ME, OV, PA, PR, RE, ST, TE, TY, UR	[104,105,106]
VDAC1 and VDAC2	The voltage-dependent anion selective channel 1 and 2	Forms a channel through the mitochondrial outer membrane and plasma membrane allowing diffusion of small hydrophilic molecules. In the plasma membrane it is involved in cell volume regulation and apoptosis. The abnormal expression or mal functioning of VDACs has been reported in multiple tumors and it has been considered as a biomarker capable of predicting treatment failure and breast cancer recurrence	BL, BR, CR, EN, GB, HN, LI, LA, LS, ME, OV, PA, PR, RE, ST, TY, UR	[107,108,109,110]

Note: This description may include information from UniProtKB, PhosphoSitePlus (v.6.5.9.3), GeneCards (Human Gene Database), and Human Protein Atlas [111,112,113]. Bladder: BL, bone (osteosarcoma): BN, breast: BR, cervical: CE, chondroblastoma: CH colon: CL, colorectal: CR, endometrial: EN, esophageal: ES, gastric: GA, gastrointestinal stromal tumor: GS glioblastoma: GB, glioma: GL, head and neck: HN, kidney: KD, laryngeal carcinoma: LC, leukemia: LE, liver: LI, lung: LU, lung adenocarcinoma: LA, lung squamous: LS, lymphoma: LY, melanoma: ME, ovarian: OV, pancreatic: PA, prostate: PR, renal: RE, skin: SK, stomach: ST, testis: TE, thyroid: TY, urothelial: UR.

## Data Availability

All generated raw files for these analyses were uploaded and are available at: http://massive.ucsd.edu/MSV000084138/ (acccessed on 5 July 2021).

## Supplementary Materials

The following are available online at https://www.mdpi.com/article/10.3390/toxins13080519/s1, Figure S1: Optical microscopy analysis of breast cancer cell lines treated with different concentrations of *B. jararaca* snake venom. MCF7 (a) and MDA-MB-231 (b) cell lines (10× magnification), Figure S2: Functional classification of the proteins presenting fold change (FC) ≥ 1.5 according to Molecular Function GO enrichment analysis. (a) MCF7 cell line. (b) MDA-MB-231 cell line, Figure S3: Functional classification of the proteins presenting FC ≥ 1.5 according to the Biological Process GO enrichment analysis. (a) MCF7 cell line. (b) MDA-MB-231 cell line, Figure S4: Functional classification of the proteins presenting FC ≥ 1.5 according to the Cellular Component GO enrichment analysis. (a) MCF7 cell line. (b) MDA-MB-231 cell line, Figure S5: Functional classification of the proteins presenting FC ≥ 1.5 according to Protein Family Classification GO enrichment analysis. (a) MCF7 cell line. (b) MDA-MB-231 cell line, Figure S6a: Protein-protein interaction of proteins identified in MCF7 cell line presenting FC ≤ 0.67 in cells treated with 2.5 μg/mL of *B. jararaca* venom compared to control, Figure S6b: Protein-protein interaction of proteins identified in MCF7 cell line presenting FC ≥ 1.5 in cells treated with 0.67 μg/mL of *B. jararaca* venom compared to control, Figure S6c: Protein-protein interaction of proteins identified in MCF7 cell line presenting FC ≤ 0.67 when in cells treated with 0.63 μg/mL of *B. jararaca* venom compared to control, Supplemental Figure S7a: Protein-protein interaction of proteins identified in MDA-MB-231 cell line presenting FC ≤ 0.67 in cells treated with 2.5 μg/mL of *B. jararaca* venom compared to control, Figure S7b: Protein-protein interaction of proteins identified in MDA-MB-231 cell line presenting FC ≤ 0.67 in cells treated with 0.63 μg/mL of *B. jararaca* venom compared to control, Figure S7c: Protein-protein interaction of proteins identified in MDA-MB-231 cell line presenting FC ≥ 2.5 in cells treated with 0.63 μg/mL of *B. jararaca* venom compared to control, Figure S8: Intensity plots of proteins identified exclusively in one or two of the conditions tested (Control, 0.63 μg/mL or 2.5 μg/mL of *B. jararaca* venom) in both MCF7 and MDA-MB-231 cell lines (Values presented in Table S4). (a) Proteins identified exclusively in the control group. (b) Proteins identified exclusively in the 0.63 μg/mL of venom treatment. (c) Proteins identified exclusively in the 2.5 μg/mL of venom treatment. (d) Proteins identified exclusively in the 0.63 and 2.5 μg/mL of venom treatments. (e) Proteins identified exclusively in the control group and 2.5 μg/mL of venom treatments. (f) Proteins identified exclusively in the control group and 0.63 μg/mL of venom treatment, Table S1a: Mass spectrometry-based proteomics analysis of MCF7 cell line treated with PBS (CTRL), 0.63 μg/mL and 2.5 μg/mL of *B. jararaca* snake venom, Table S2a: Mass spectrometry-based proteomics analysis of MCF7 cell lines treated with PBS (CTRL), 0.63 mg/mL and 2.5 μg/mL of *B. jararaca* snake venom. Fold change based on the control LFQ intensity, Table S1b: Mass spectrometry-based proteomics analysis of MDA-MB-231 cell line treated with PBS (CTRL), 0.63 μg/mL, and 2.5 μg/mL of *B. jararaca* snake venom, Table S2b: Mass spectrometry-based proteomics analysis of MDA-MB-231 cell lines treated with PBS (CTRL), 0.63 μg/mL and 2.5 μg/mL of *B. jararaca* snake venom. Fold change based on the control LFQ intensity, Table S3: Protein list of each of the seven clusters corresponding to Figure 3, Table S4a: MCF7 proteins identified exclusively at one or two of the conditions tested: PBS (CTRL), 0.63 μg/mL and 2.5 μg/mL of *B. jararaca* snake venom, Table S4b: MDA-MB-231 proteins identified exclusively at one or two of the conditions tested: PBS (CTRL), 0.63 μg/mL and 2.5 μg/mL of *B. jararaca* snake venom.
