# Supplementary material for: Bothrops Jararaca Snake Venom Modulates Key Cancer-Related Proteins in Breast Tumor Cell Lines
Source: Toxins (Basel). 2021 Jul 25;13(8):519. doi: 10.3390/toxins13080519 (PMC8402457; doi:10.3390/toxins13080519)
Supplement: Supplementary file 1 [file toxins-13-00519-s001.zip › toxins-1308565-supplementary/toxins-1308565/Supplemental Figures S1 - S8.pdf]

Supplementary Materials: Bothrops Jararaca Snake Venom Modulates Key Cancer-Related Proteins in Breast Tumor Cell Lines

Carolina Yukiko Kisaki, Stephanie Santos Suehiro Arcos, Fabio Montoni, Wellington da Silva Santos, Hamida Macêdo Calacina, Ismael Feitosa Lima, Daniela Cajado-Carvalho, Emer Suavinho Ferro, Milton Yutaka Nishiyama-Jr and Leo Kei Iwai

(a)

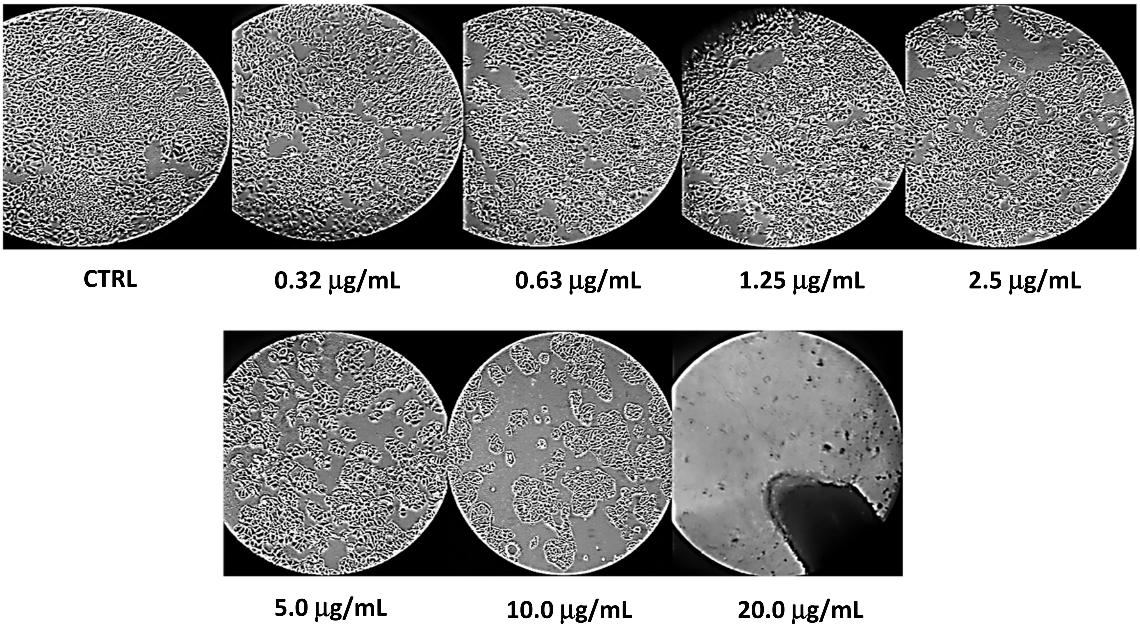

(b)

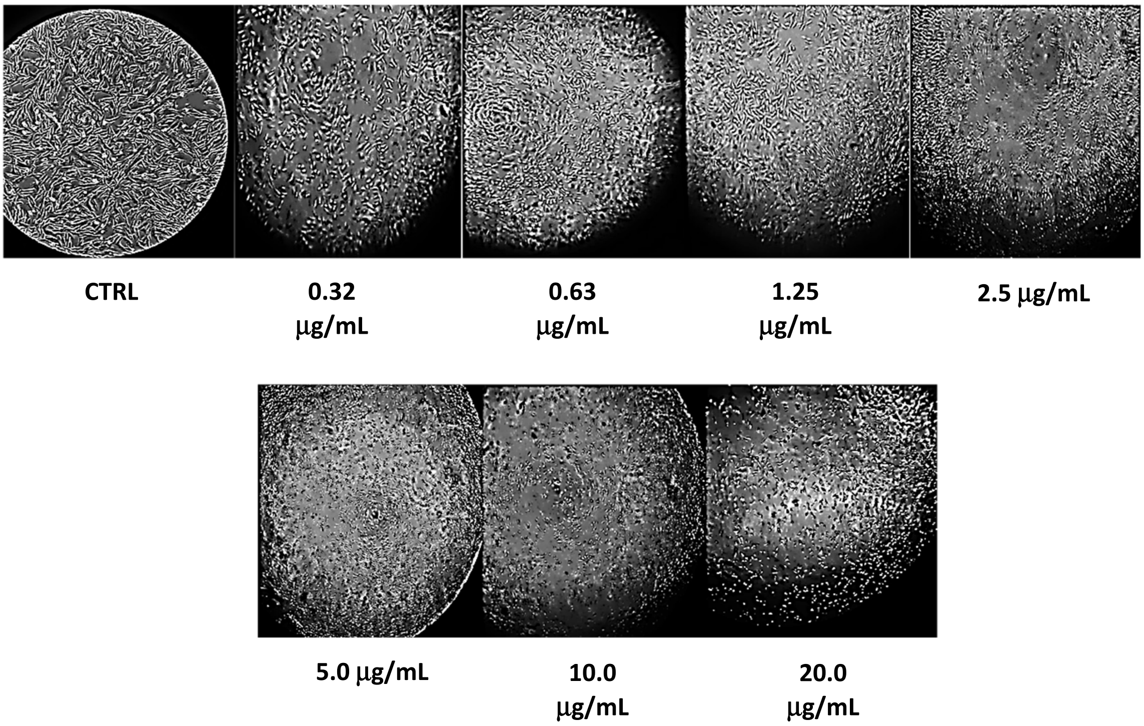

Figure S1. Optical microscopy analysis of breast cancer cell lines treated with different concentrations of *B. jararaca* snake venom. MCF-7 (a) and MDA-MB-231 (b) cell lines (10x magnification).

(a)

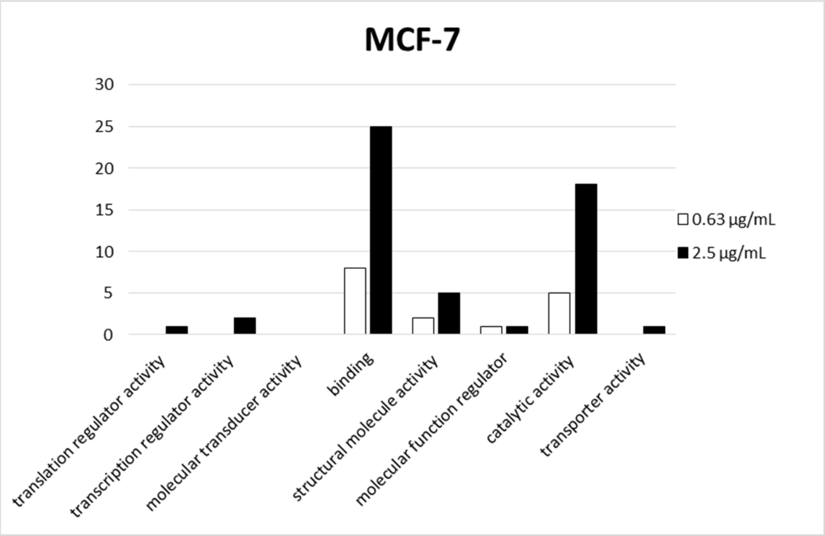

(b)

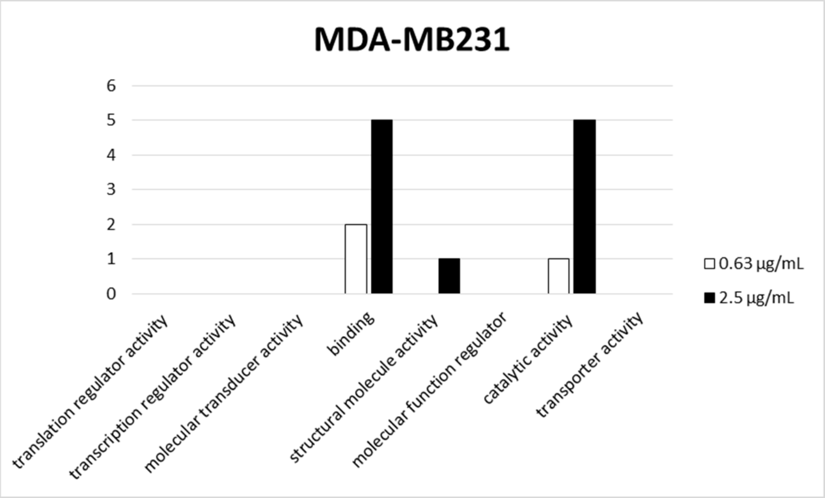

Figure S2. Functional classification of the proteins presenting fold change (FC) ≥ 1.5 according to Molecular Function GO enrichment analysis. (a) MCF-7 cell line. (b) MDA-MB-231 cell line.

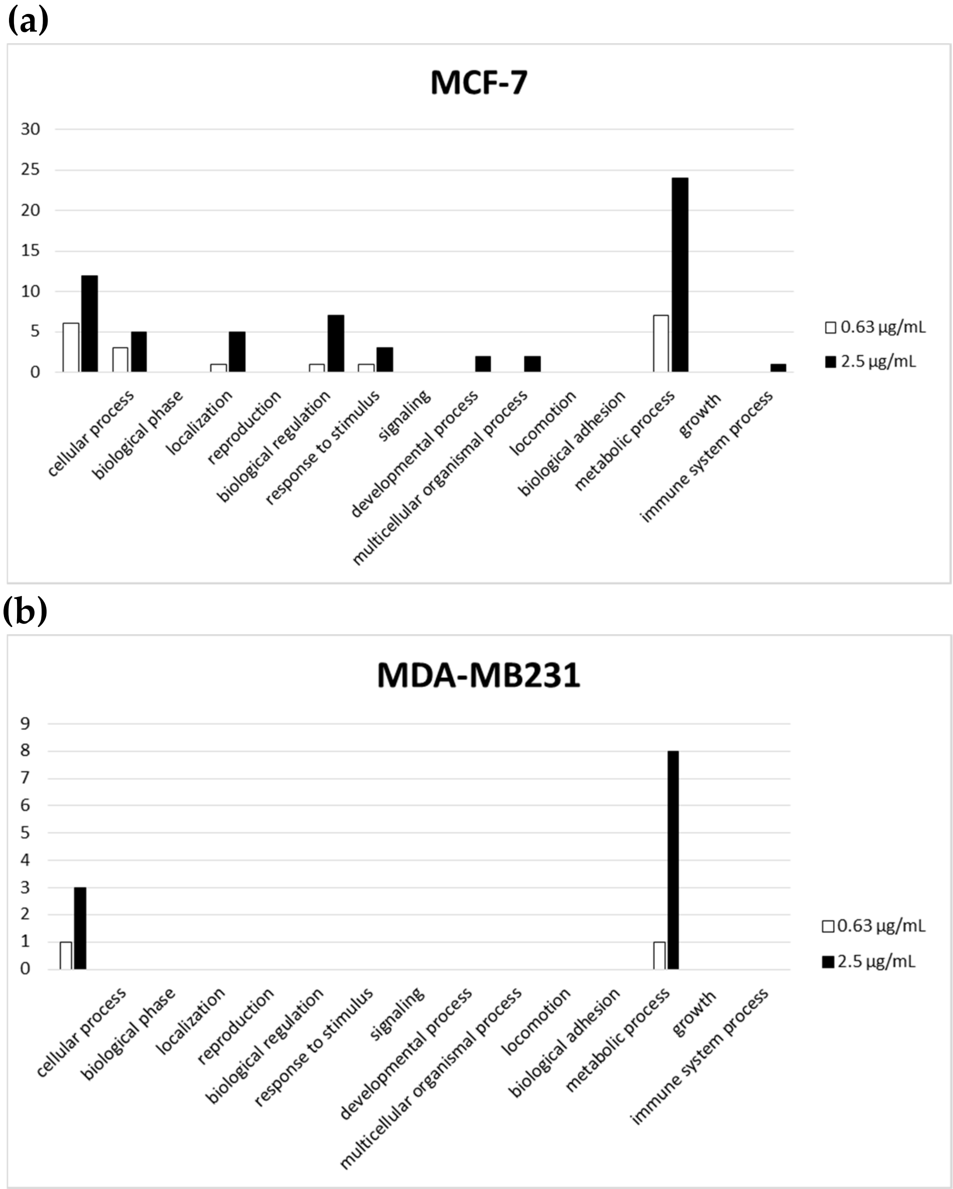

Figure S3. Functional classification of the proteins presenting FC ≥ 1.5 according to the Biological Process GO enrichment analysis. (a) MCF-7 cell line. (b) MDA-MB-231 cell line.

(a)

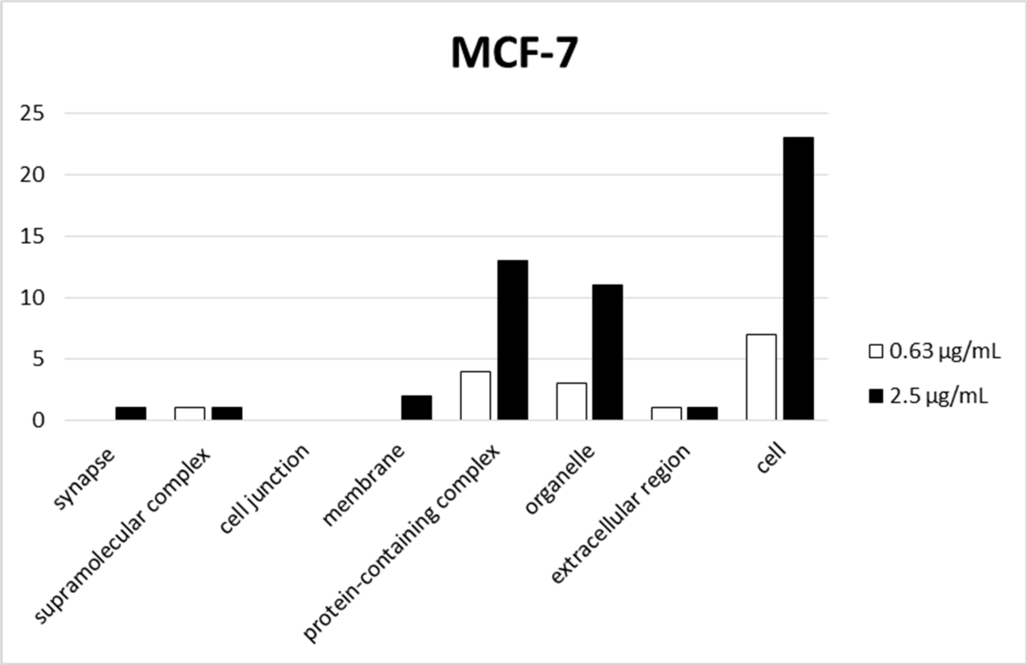

(b)

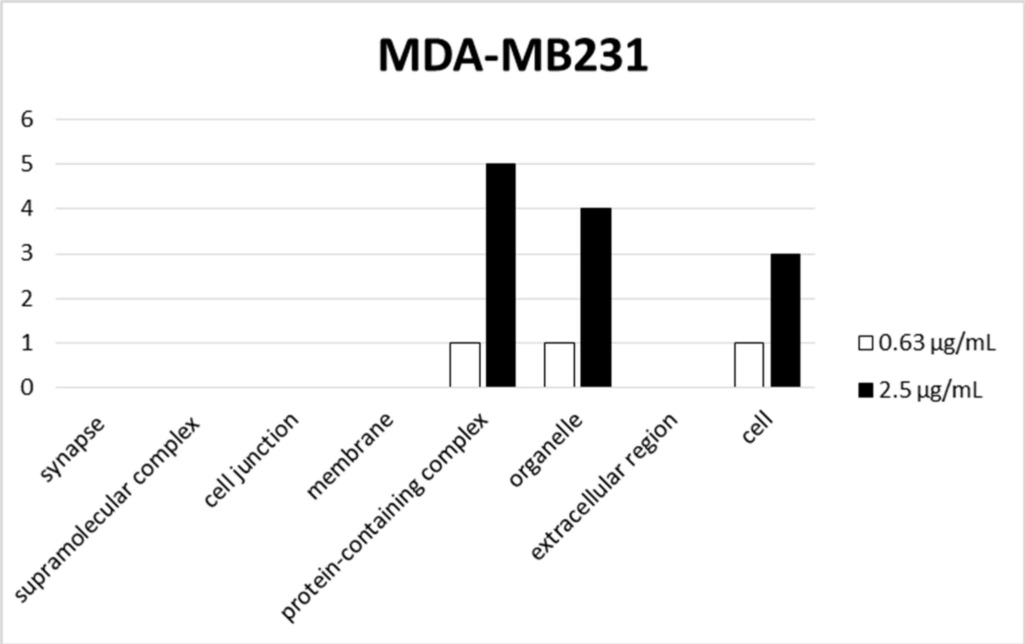

Figure S4. Functional classification of the proteins presenting FC ≥ 1.5 according to the Cellular Component GO enrichment analysis. (a) MCF-7 cell line. (b) MDA-MB-231 cell line.

(a)

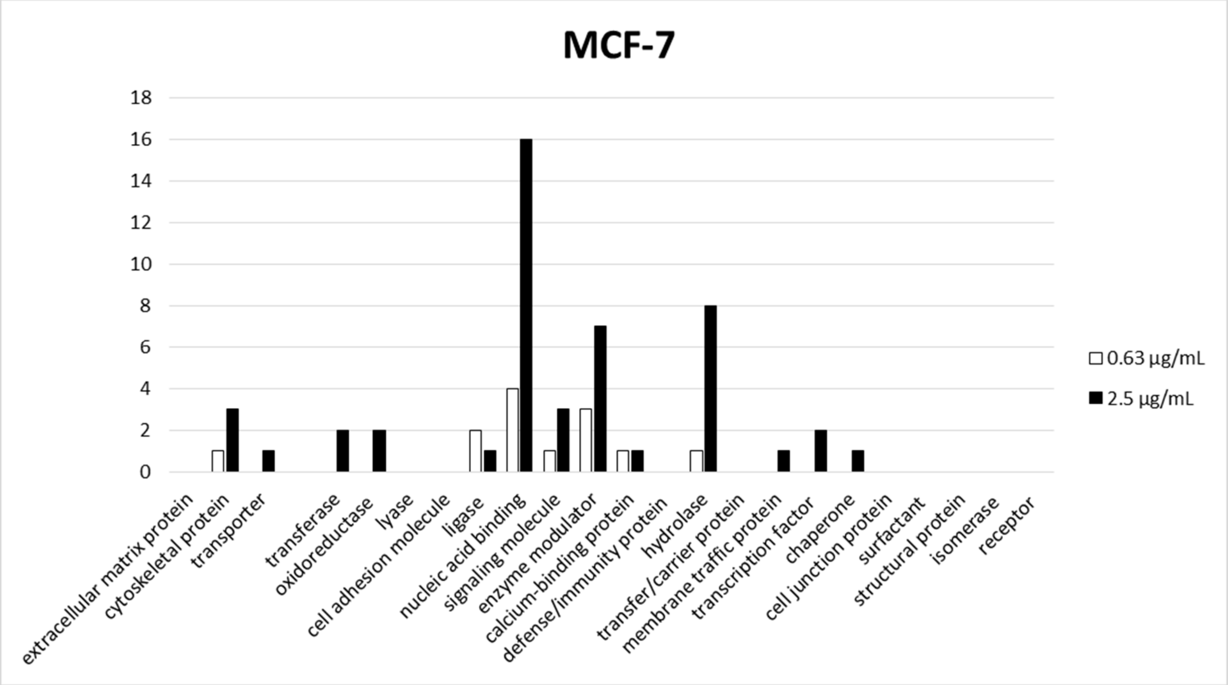

(b)

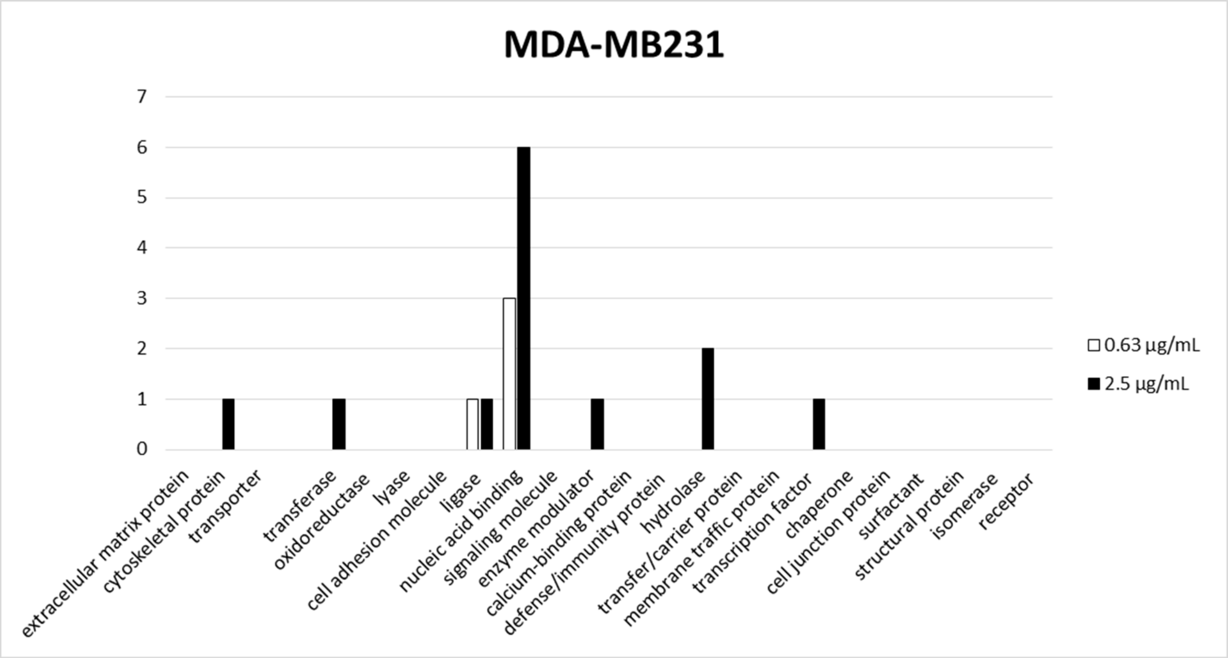

Figure S5. Functional classification of the proteins presenting FC ≥ 1.5 according to Protein Family Classification GO enrichment analysis. (a) MCF-7 cell line. (b) MDA-MB-231 cell line.

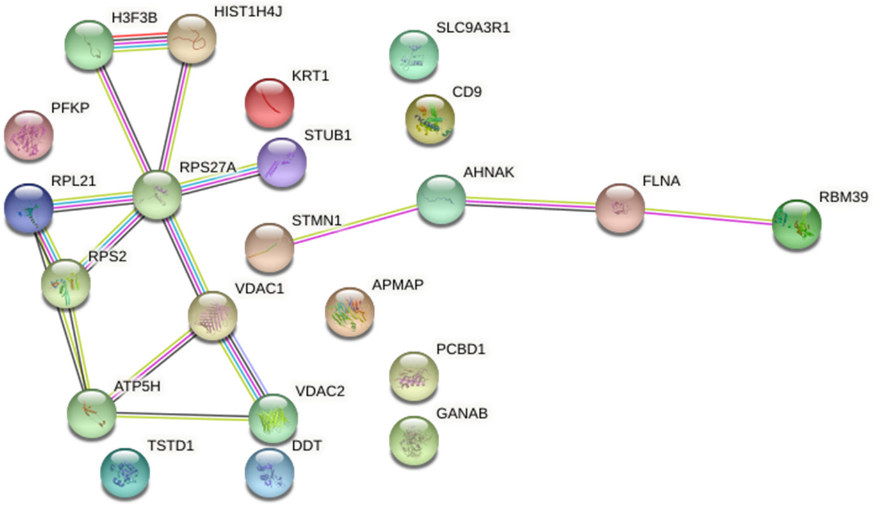

Figure S6. a. Protein-protein interaction of proteins identified in MCF-7 cell line presenting FC ≤ 0.67 in cells treated with 2.5 g/mL of *B. jararaca* venom compared to control.

Number of nodes: 22, number of edges: 16, average node degree: 1.45, avg. local clustering coefficient: 0.37, expected number of edges: 8, PPI enrichment p-value: 0.0107.

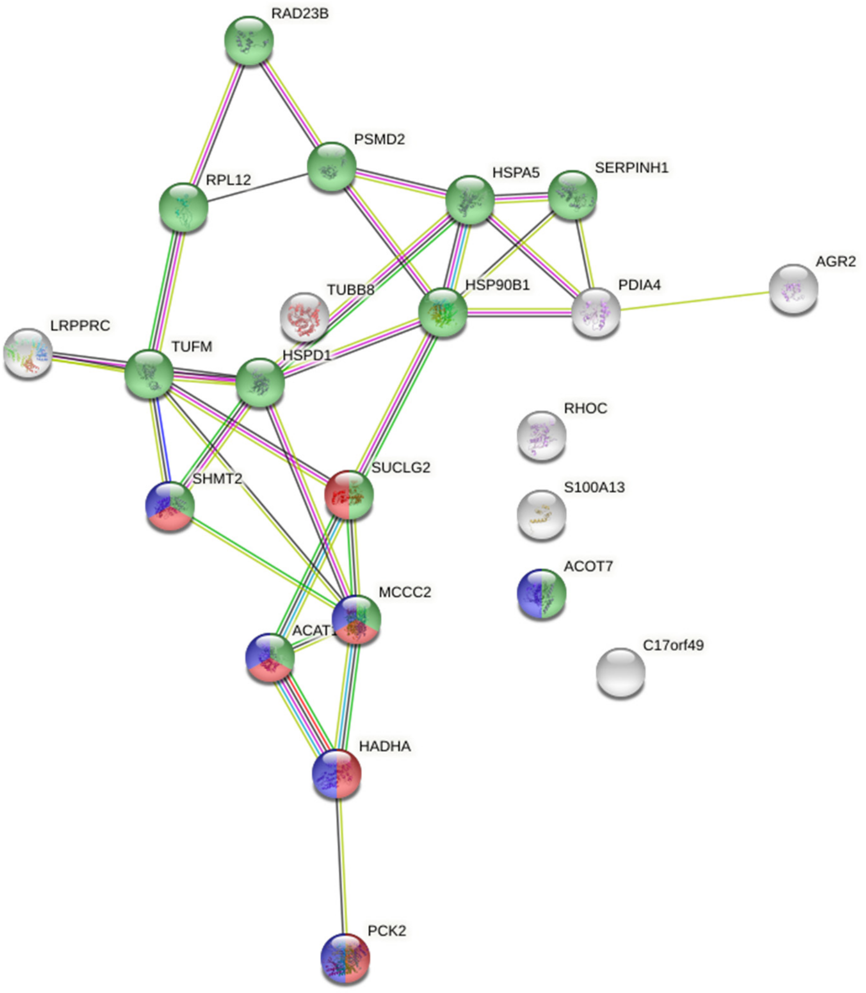

Figure S6. b. Protein-protein interaction of proteins identified in MCF-7 cell line presenting FC ≥ 1.5 in cells treated with 0.67 g/mL of *B. jararaca* venom compared to control.

Red: metabolism pathway (Reactome). Blue: metabolic pathways (KEGG). Green: organonitrogen compound metabolic process (GO Biological Process). Number of nodes: 22, number of edges: 31, average node degree: 2.82, avg. local clustering coefficient: 0.473, expected number of edges: 6, PPI enrichment p-value: 2.28e-12.

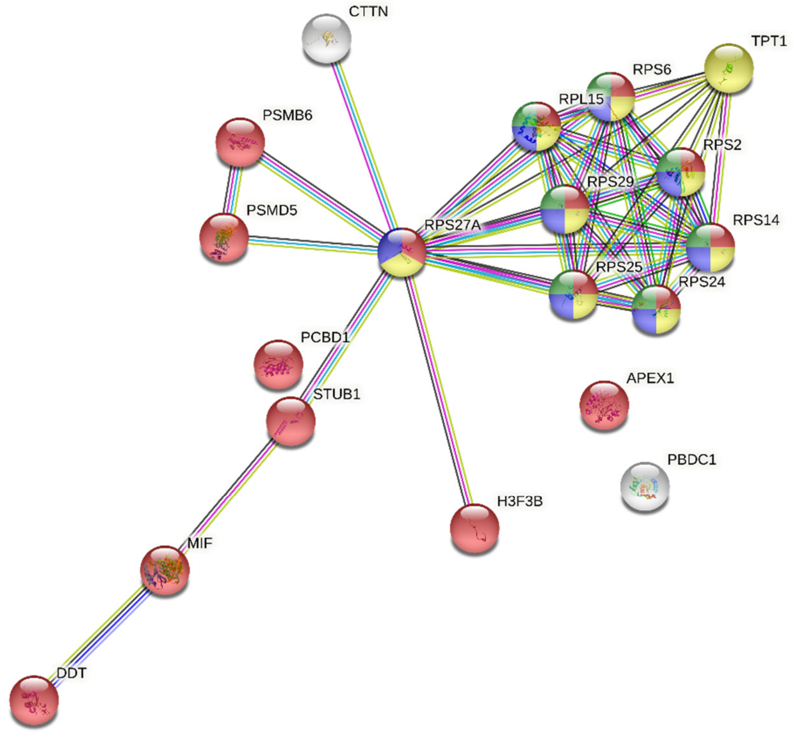

Figure S6. c. Protein-protein interaction of proteins identified in MCF-7 cell line presenting FC ≤ 0.67 when in cells treated with 0.63 g/mL of *B. jararaca* venom compared to control.

Red: Cellular protein metabolic process (GO, Biological process). Blue: Ribosome (KEGG pathways). Green: Developmental Biology (Reactome Pathways). GTP hydrolysis and joining of the 60S ribosomal subunit (Local network cluster, STRING). Number of nodes: 19, number of edges: 44, average node degree: 4.63, avg. local clustering coefficient: 0.704, expected number of edges: 14, PPI enrichment p-value: 5.2e-11

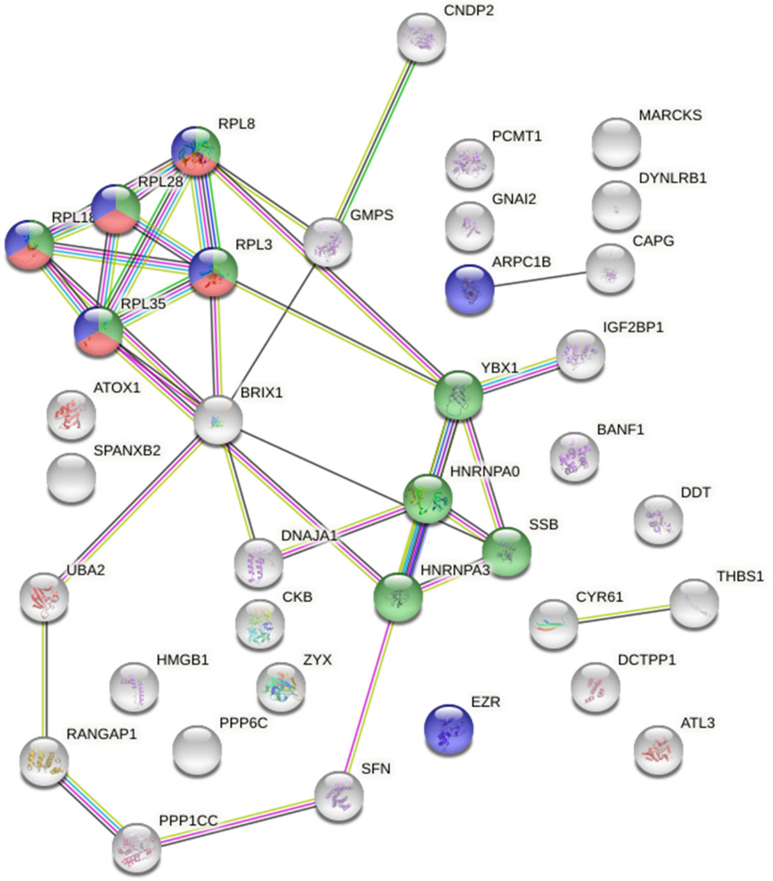

Figure S7. a. Protein-protein interaction of proteins identified in MDA-MB-231 cell line presenting FC ≤ 0.67 in cells treated with 2.5 g/mL of *B. jararaca* venom compared to control.

Red: Ribosome (KEGG Pathways). Blue: Developmental Biology (Reactome Pathways). Green: mRNA metabolic process (GO Biological Process). Number of nodes: 37, number of edges: 36, average node degree: 1.95, avg. local clustering coefficient: 0.3, expected number of edges: 18, PPI enrichment p-value: 0.000181

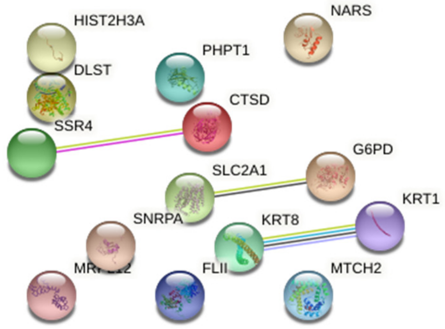

Figure S7. b. Protein-protein interaction of proteins identified in MDA-MB-231 cell line presenting FC ≤ 0.67 in cells treated with 0.63 g/mL of *B. jararaca* venom compared to control.

Number of nodes: 14, number of edges: 3, average node degree: 0.429, avg. local clustering coefficient: 0.429, expected number of edges: 2, PPI enrichment p-value: 0.201.

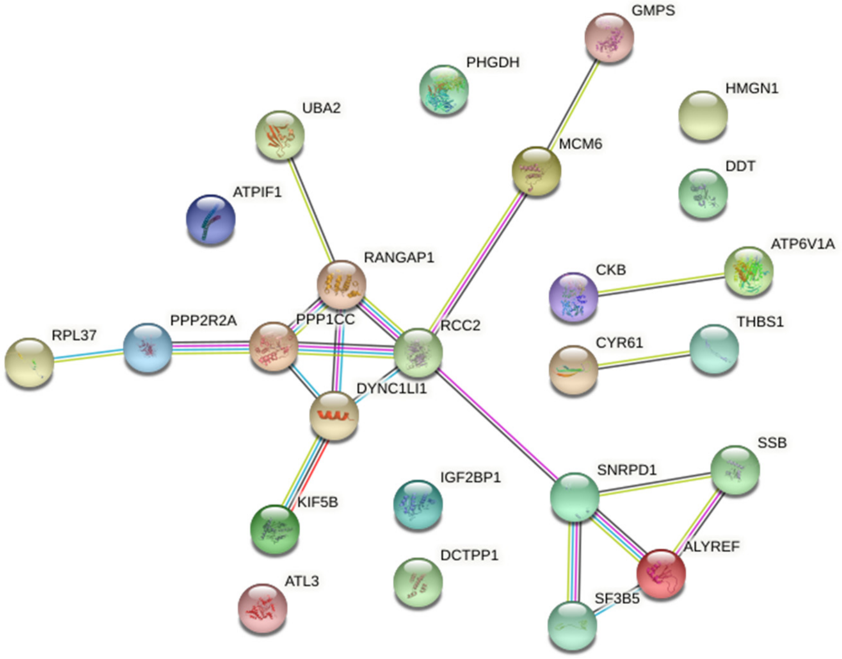

Figure S7. c. Protein-protein interaction of proteins identified in MDA-MB-231 cell line presenting FC ≥ 2.5 in cells treated with 0.63 g/mL of *B. jararaca* venom compared to control.

Number of nodes: 25, number of edges: 20, average node degree: 1.6, avg. local clustering coefficient: 0.512, expected number of edges: 8, PPI enrichment p-value: 0.000321.

(a)

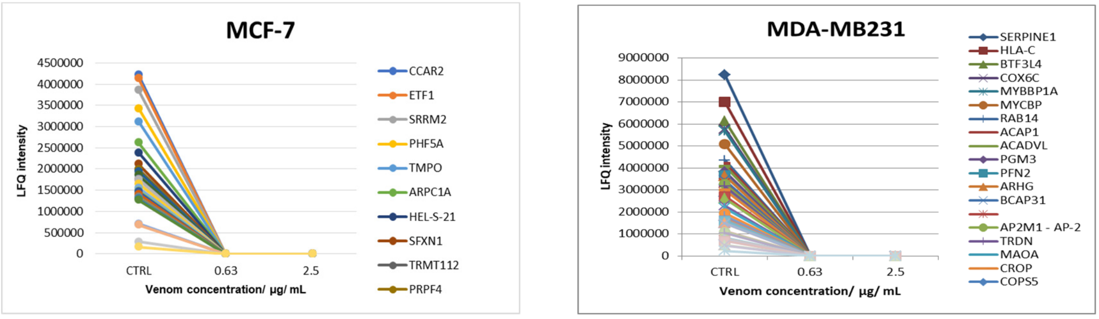
MCF-7
LFQ intensity
Venom concentration/ µg/ mL
CTRL
0.63
2.5
CCAR2
ETF1
SRRM2
PHF5A
TMPO
ARPC1A
HEL-S-21
SFXN1
TRMT112
PRPF4
MDA-MB231
LFQ intensity
Venom concentration/ µg/ mL
SERPINE1
HLA-C
BTF3L4
COX6C
MYBBP1A
MYCBP
RAB14
ACAP1
ACADVL
PGM3
PFN2
ARHG
BCAP31
AP2M1 - AP-2
TRDN
MAOA
CROP
COPS5

(b)

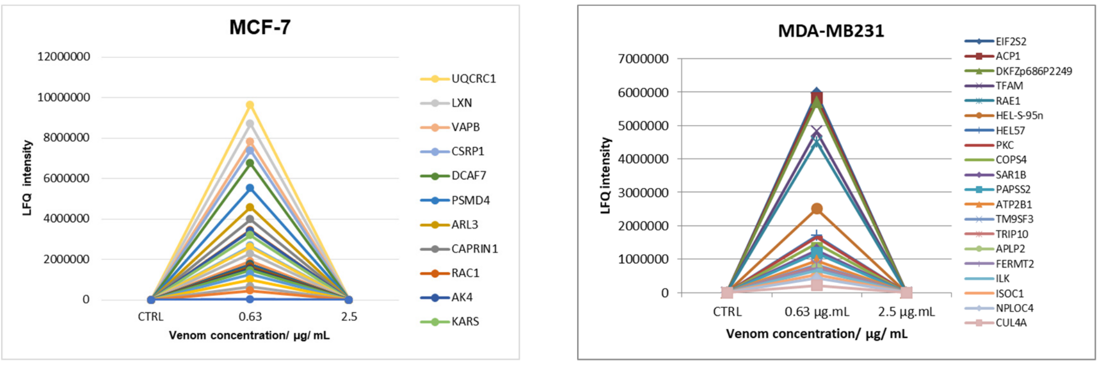
MCF-7
LFQ intensity
Venom concentration/ µg/ mL
CTRL
0.63
2.5
UQCRC1
LXN
VAPB
CSRP1
DCAF7
PSMD4
ARL3
CAPRIN1
RAC1
AK4
KARS
MDA-MB231
LFQ intensity
Venom concentration/ µg/ mL
CTRL
0.63 µg.mL
2.5 µg.mL
EIF2S2
ACP1
DKFZp686P2249
TFAM
RAE1
HEL-S-95n
HEL57
PKC
COPS4
SAR1B
PAPSS2
ATP2B1
TM9SF3
TRIP10
APLP2
FERMT2
ILK
ISOC1
NPLOC4
CUL4A

(c)

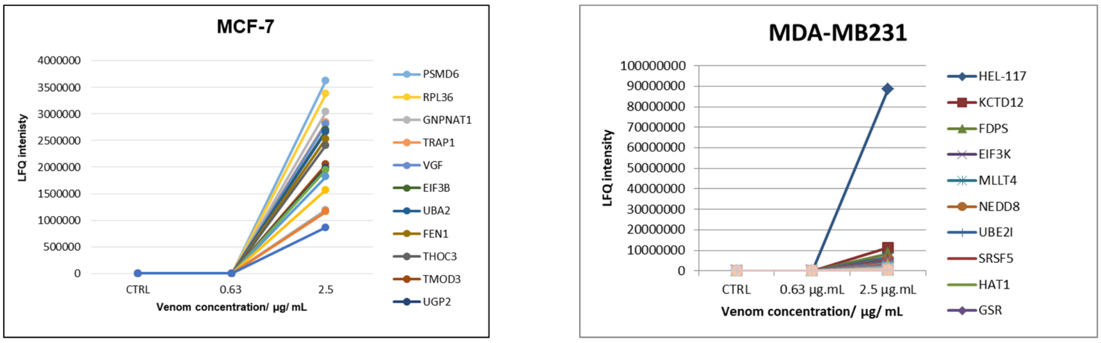
MCF-7
LFQ intenisty
Venom concentration/ µg/ mL
CTRL
0.63
2.5
PSMD6
RPL36
GNPNAT1
TRAP1
VGF
EIF3B
UBA2
FEN1
THOC3
TMOD3
UGP2
MDA-MB231
LFQ intensity
Venom concentration/ µg/ mL
CTRL
0.63 µg.mL
2.5 µg.mL
HEL-117
KCTD12
FDPS
EIF3K
MLLT4
NEDD8
UBE2I
SRSF5
HAT1
GSR

(d)

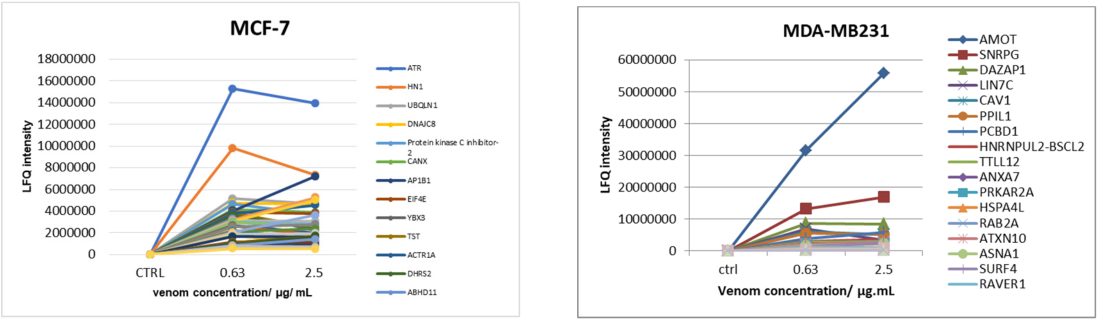
MCF-7
LFQ intensity
venom concentration/ µg/ mL
CTRL
0.63
2.5
ATR
HN1
UBQLN1
DNAJC8
Protein kinase C inhibitor-2
CANX
AP1B1
EIF4E
YBX3
TST
ACTR1A
DHRS2
ABHD11
MDA-MB231
LFQ intensity
Venom concentration/ µg.mL
ctrl
0.63
2.5
AMOT
SNRPG
DAZAP1
LIN7C
CAV1
PPIL1
PCBD1
HNRNPUL2-BSCL2
TTLL12
ANXA7
PRKAR2A
HSPA4L
RAB2A
ATXN10
ASNA1
SURF4
RAVER1

(e)

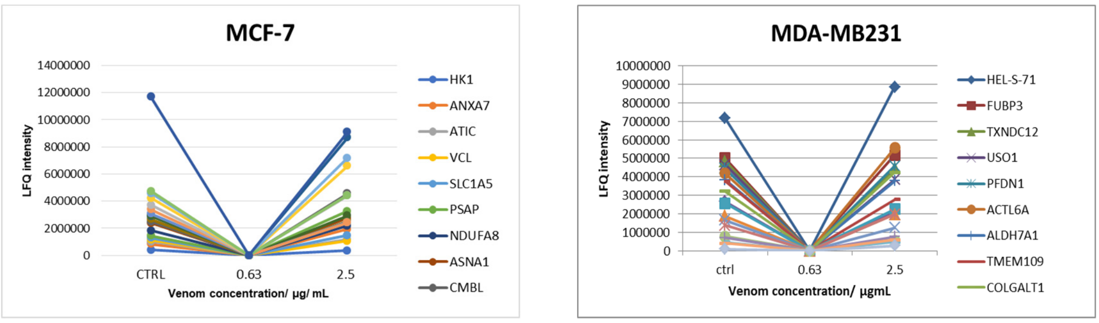

(f)

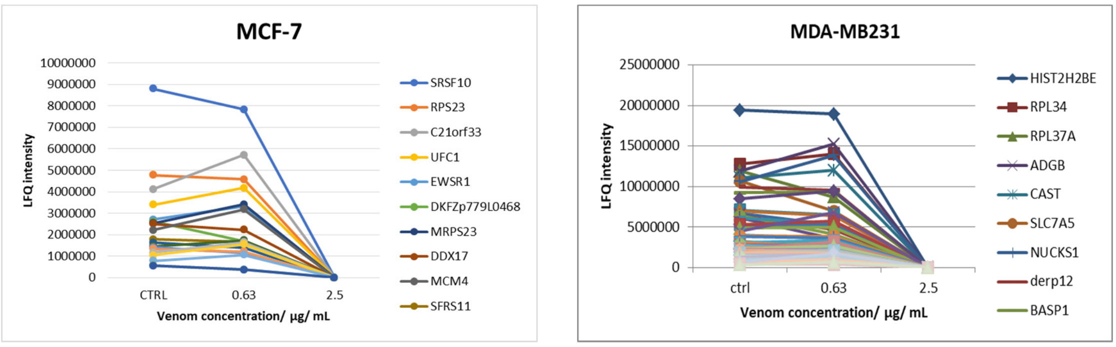

Figure S8. Intensity plots of proteins identified exclusively in one or two of the conditions tested (Control, 0.63 g/mL or 2.5 g/mL of *B. jararaca* venom) in both MCF7 and MDA-MB-231 cell lines (Values presented in Supplemental Table S4). **(a)** Proteins identified exclusively in the control group. **(b)** Proteins identified exclusively in the 0.63 g/mL of venom treatment. **(c)** Proteins identified exclusively in the 2.5 g/mL of venom treatment. **(d)** Proteins identified exclusively in the 0.63 and 2.5 g/mL of venom treatments. **(e)** Proteins identified exclusively in the control group and 2.5 g/mL of venom treatments. **(f)** Proteins identified exclusively in the control group and 0.63 g/mL of venom treatment.
